# Multiple electrolyte imbalances in hospitalized patients: a multimorbidity perspective from a large, retrospective cohort study

**DOI:** 10.1080/07853890.2026.2618318

**Published:** 2026-02-06

**Authors:** Nan Jiang, Siyu Liang, Yuelun Zhang, Lize Sun, Shi Chen, Hui Pan

**Affiliations:** a Key Laboratory of Endocrinology of National Health Commission, Department of Endocrinology, Peking Union Medical College Hospital, Chinese Academy of Medical Sciences and Peking Union Medical College, Beijing, China; b Department of Radiation Oncology, National Cancer Center/National Clinical Research Center for Cancer/Cancer Hospital, Chinese Academy of Medical Sciences and Peking Union Medical College, Beijing, China; c Peking Union Medical College Hospital, Chinese Academy of Medical Sciences & Peking Union Medical College, Medical Research Center, Beijing, China; d Eight-year Program of Clinical Medicine, Peking Union Medical College Hospital, Peking Union Medical College, Chinese Academy of Medical Sciences, Beijing, China

**Keywords:** Water-electrolyte imbalance, multimorbidity, prevalence, prognosis

## Abstract

**Introduction:**

Multiple electrolyte imbalances (MEIs) are underexplored in hospitalized patients. We aimed to determine: (1) the prevalence and prognostic impact of MEIs; (2) the associations between electrolyte imbalance (EI) combinations and adverse outcomes; and (3) the potential interactions among EI types.

**Materials and methods:**

Hospitalized patients at Peking Union Medical College Hospital were enrolled from 2015 to 2020. Adverse outcomes included in-hospital mortality or discharge against medical advice. Multivariable logistic regression models were used to evaluate the associations of number of EIs, EI types, and their combinations with adverse outcomes and to calculate the population-attributable fractions (PAFs). The additive and multiplicative interactions were examined for each EI combination.

**Results:**

Among 324,056 hospitalizations, the prevalence of MEIs was 18.8%. Compared to patients without EIs, the odds ratios (ORs) for adverse outcomes were 2.18 (95% confidence interval [CI]: 1.83–2.59) for patients with 1 EI and 17.34 (95% CI: 15.32–19.62) for those with ≥2 EIs. The highest-risk EI combinations at the individual level were hypercalcemia-hypernatremia (OR = 14.96 [95% CI: 11.34–19.68]), hyponatremia-hypernatremia (13.00 [95% CI: 10.09–16.74]), and hypochloremia-hypernatremia (11.06 [95% CI: 8.51–14.34]), while hypokalemia-hypernatremia (PAF = 17.89% [95% CI: 17.37%-18.41%]), hyperchloremia-hypernatremia (15.29% [95% CI: 14.99%–15.59%]), and hypocalcemia-hypernatremia (12.40% [95% CI: 11.94%–12.86%]) contributed the most to adverse outcomes at the population level. Synergistic additive interactions were observed for hyperchloremia-hypernatremia (relative excess risk due to interaction = 4.80 [95% CI: 3.05–6.55]) and hypercalcemia-hypernatremia (6.98 [95% CI: 3.11–10.86]).

**Conclusions:**

MEIs are common and harmful in hospitalized patients. Prioritizing different intervention targets at individual and population levels may improve clinical outcomes.

## Introduction

Electrolytes are widely distributed both inside and outside human cells. Their concentrations are precisely regulated to preserve cellular integrity and function, thereby supporting diverse metabolic and physiological processes. Electrolyte imbalances (EIs) refer to deviations from normal serum electrolyte levels, which can be caused by various factors such as diseases, medications, high temperature, improper diet, and excessive exercise [1]. Thus, EIs are common not only among hospitalized patients but also in the general population [2]. The detrimental impact of EIs are well recognized, including prolonged hospital stays, increased mortality, and higher intensive care unit admission rates [3–5].

Multiple electrolyte imbalances (MEIs), the occurrence of two or more EI types in the same patient, are not rare in clinical practice. A study from a Norwegian emergency department showed that the proportions of patients with no EIs, 1 EI, 2 EIs, and >2 EIs were 37.4%, 34.2%, 17.3%, and 11.0%, respectively; as number of EIs increased, so did the patients’ hospital length of stay, readmission risk, and mortality risk [4]. Another study among cancer patients in Shanghai reported that 38.8%, 28.2%, and 33.0% of patients experienced 1, 2, and >2 EIs, respectively, with a gradient increase in in-hospital mortality risk [5]. These findings highlight that the diversity and complexity of EIs have a significant impact on the prognosis of hospitalized patients.

Despite extensive research on EIs, most studies have focused on specific electrolytes (such as sodium and potassium), with limited attention given to anions like chloride. Furthermore, research subjects are often restricted to specific disease populations (e.g. patients with heart failure or COVID-19) [6,7] or risk groups (e.g. patients receiving immune checkpoint inhibitors or emergency patients) [4,8], which limits the generalizability of the findings. Another important limitation of current literature is its predominant focus on the independent prognostic effects of different EI types. This approach carries two key shortcomings: first, the estimated effect of a single EI type may be inflated if other coexisting EIs are not adequately adjusted for; second, treating EI types in isolation overlooks potential interactions among different EIs and fails to capture their combined influence on patient outcomes, thereby reducing clinical translatability. Although some studies have begun to compare outcomes among patients with isolated hyponatremia, isolated hypernatremia, and mixed dysnatremia [9,10], these investigations remain restricted to sodium and do not examine its interplay with other electrolytes. In summary, despite being a common endocrine and metabolic disturbance, MEIs remain understudied. Epidemiological data and prognostic analyses in the general inpatient population are particularly scarce, highlighting a notable gap in current medical knowledge.

With an aging population and the rising prevalence of chronic diseases, multimorbidity – the coexistence of two or more diseases in an individual – has gained increasing recognition as a critical medical and public health challenge [11,12]. The understanding of multimorbidity has evolved from viewing it as a simple aggregation of diseases to recognizing that synergistic interactions among coexisting conditions may amplify their collective harm, leading to worse health outcomes than expected from each disease alone [13].

In parallel, MEIs can be viewed as a specific metabolic subtype of multimorbidity. We hypothesize that MEIs may not merely reflect the additive burden of individual electrolyte disturbances, but could involve interactive effects that modify their overall clinical impact, resulting in more complex and severe prognostic consequences. Building on the conceptual framework of multimorbidity, we introduce the novel concept of ‘EI combinations’. By systematically examining paired EI types, we aim to uncover common patterns of co-occurrence, quantify their interactive effects, and evaluate their joint prognostic significance among general hospitalized patients. This innovative perspective seeks to shift the clinical and research paradigm from a conventional, single-electrolyte focus toward an integrated, multi-electrolyte perspective, ultimately paving the way for more holistic patient management and future investigative directions in electrolyte disorders.

Based on a large sample of adult hospitalizations, this study focuses on four common electrolytes – sodium, potassium, calcium, and chloride – and adopts an innovative multimorbidity perspective. Our objectives are to: (1) determine the prevalence of MEIs and their impact on prognosis among hospitalized patients; (2) quantify the associations between different EI combinations and adverse outcomes at both the individual and population levels; and (3) identify additive and multiplicative interactions among different EI types.

## Materials and methods

### Study design, subjects, and ethics approval

This retrospective cohort study collected hospitalization data from Peking Union Medical College Hospital between January 1, 2015, and August 11, 2020. Data were obtained from the Electronic Medical Record System, including patient ID number, age, sex, diagnoses, laboratory test results, and discharge status. All patient ID numbers were anonymized prior to analysis. The exclusion criteria were as follows: (1) age under 18 years; (2) unclear discharge status; and (3) no serum sodium, potassium, calcium, or chloride measurements during hospitalization.

This study was approved by the Ethics Committee of Peking Union Medical College Hospital (approval no. I-23PJ405, approval date 03/09/2023) and conducted in accordance with the Declaration of Helsinki. As this was a retrospective study using anonymized clinical data, the requirement for informed consent was waived by the committee.

### Exposure and outcome definitions

Diagnoses were coded according to the International Classification of Diseases, 10th Revision (ICD-10) and categorized into 19 comorbidity groups: myocardial infarction, congestive heart failure, peripheral vascular disease, central vascular disease, dementia, chronic lung disease, connective tissue disease, peptic ulcer, mild liver disease, diabetes without complications, diabetes with complications, hemiplegia, moderate to severe kidney disease, tumor without distant metastasis, leukemia, lymphoma, moderate to severe liver disease, metastatic solid tumor, and acquired immunodeficiency syndrome. Based on these groups, the Charlson comorbidity index (CCI) was calculated to assess overall comorbidity burden (Table S1) [14].

Serum electrolyte levels during hospitalization were defined as all values measured from admission to discharge. To account for the effect of hyperglycemia on sodium levels, the following correction formula was used: corrected sodium (mmol/L) = measured sodium (mmol/L) + 2.4 × (glucose [mmol/L] − 5.5)/5.5 [15]. Calcium levels were corrected based on simultaneously measured albumin levels using the formula: corrected calcium (mmol/L) = measured calcium (mmol/L) + 0.02 × (40 – albumin [g/L]) [16]. The reference ranges were defined as follows: sodium 135–145 mmol/L; potassium 3.5–5.5 mmol/L; calcium 2.13–2.70 mmol/L; chloride 96–111 mmol/L. Electrolyte levels below or above these thresholds were categorized as hypo-EIs or hyper-EIs, respectively. A total of eight EI types were analyzed: hyponatremia, hypernatremia, hypokalemia, hyperkalemia, hypocalcemia, hypercalcemia, hypochloremia, and hyperchloremia. The cutoff values for defining severe cases of the first six EI types were based on established thresholds from the literature [17–22]. For severe hypochloremia and hyperchloremia, for which no standard definitions exist, we defined the severe cases as the first and third tertiles of the hypochloremic and hyperchloremic chloride values, respectively [23] (Table 1). For patients with multiple electrolyte measurements, the presence of any abnormal value during hospitalization was considered sufficient to diagnose the corresponding EI (e.g. if serum sodium <135 mmol/L at any point, the patient was classified as having hyponatremia). The number of EIs during hospitalization was recorded and categorized as 0, 1, or ≥2. Based on the eight EI types, there were 28 possible EI combinations.

**Table 1.** Normal serum electrolyte levels and the severe EI types.

Definition	Range
Normonatremia	135–145 mmol/L
Severe hyponatremia	<125 mmol/L [17]
Severe hypernatremia	>155 mmol/L [18]
Normokalemia	3.5–5.5 mmol/L
Severe hypokalemia	<2.5 mmol/L [19]
Severe hyperkalemia	>6.5 mmol/L [20]
Normocalcemia	2.13–2.70 mmol/L
Severe hypocalcemia	<1.88 mmol/L [21]
Severe hypercalcemia	>3.50 mmol/L [22]
Normochloremia	96–111 mmol/L
Severe hypochloremia	<92 mmol/L [23]
Severe hyperchloremia	>114 mmol/L [23]

Our primary outcome was a composite of in-hospital mortality and discharge against medical advice. Although biologically distinct, both endpoints signify a clinically adverse hospitalization. In-hospital death is self-evident; discharge against medical advice in our setting typically reflects a terminal prognosis, as it often follows a family’s decision to withdraw active treatment for conditions deemed irrecoverable and is associated with poor post-discharge outcomes [24]. We therefore combined them to capture the broader construct of a ‘failed hospitalization’, collectively termed adverse outcomes.

### Statistical analysis

For continuous variables, normally and non-normally distributed data were presented as mean ± standard deviation and median (1st quartile, 3rd quartile), respectively. Categorical variables were expressed as frequency (percentage). Trend tests were conducted to assess baseline characteristics across varying numbers of EIs.

For each EI combination, a univariate logistic regression model was used to assess the association between the two EI types, with the tendency for co-occurrence quantified by the odds ratio (OR) and its Bonferroni-corrected 95% confidence interval (CI). For individual-level risk assessment, multivariable logistic regression models were applied to estimate the ORs and 95% CIs for the associations of number of EIs, EI types, and EI combinations with adverse outcomes. Bonferroni correction was applied to the 95% CIs for EI types and their combinations. The models were adjusted for age, sex, CCI, myocardial infarction, congestive heart failure, peripheral vascular disease, cerebrovascular disease, moderate to severe renal disease, diabetes, chronic pulmonary disease, and moderate to severe liver disease. When evaluating the association of a specific EI type or EI combination with adverse outcomes, the model additionally adjusted for the presence of other EI types (e.g. when assessing the prognostic impact of hyponatremia and hyponatremia–hypernatremia, the other seven and six EI types were included as covariates, respectively). Firth’s penalized maximum likelihood estimation method was employed to reduce potential bias due to sparse data, and patient ID number was included as a random effect to account for correlation from repeated hospitalizations. Variance inflation factors were examined to confirm the absence of multicollinearity among covariates.

For population-level risk assessment, after excluding repeated hospitalizations (retaining only the first hospitalization record for each patient), we computed the population-attributable fractions (PAFs) and their Bonferroni-adjusted 95% CIs. PAF estimates the proportion of adverse outcomes that could be prevented if the risk factor (i.e. a specific EI type or EI combination) is absent, reflecting its contribution to the primary outcome at the population level.

Additive and multiplicative interactions among different EI types were evaluated. For additive interactions, we calculated the relative excess risk due to interaction (RERI), attributable proportion (AP), and synergy index (SI), along with their Bonferroni-corrected 95% CIs. A positive RERI indicates that the combined risk of the two EI types exceeds the sum of their individual risks, while a negative RERI suggests the opposite. AP and SI served as sensitivity analyses, where AP ≠ 0 or SI ≠ 1 implies evidence of interaction. For multiplicative interactions, we calculated the interaction term’s OR and its Bonferroni-corrected 95% CI. An OR > 1 indicates that the combined risk of the two EI types exceeds the product of their individual risks.

To explore potential heterogeneity across diagnostic categories, stratified analyses were performed among patients with cancer (tumor with or without metastasis), heart disease (myocardial infarction or congestive heart failure), moderate to severe liver disease, and moderate to severe renal disease. For sensitivity analyses, models were further adjusted for care setting (intensive care unit or general ward). Models were also refitted by excluding discharge against medical advice from the primary outcome.

A two-sided p-value < 0.05 was considered statistically significant. All analyses were performed using R software, version 4.2.3.

## Results

### Characteristics of the study subjects

**Figure 1.**
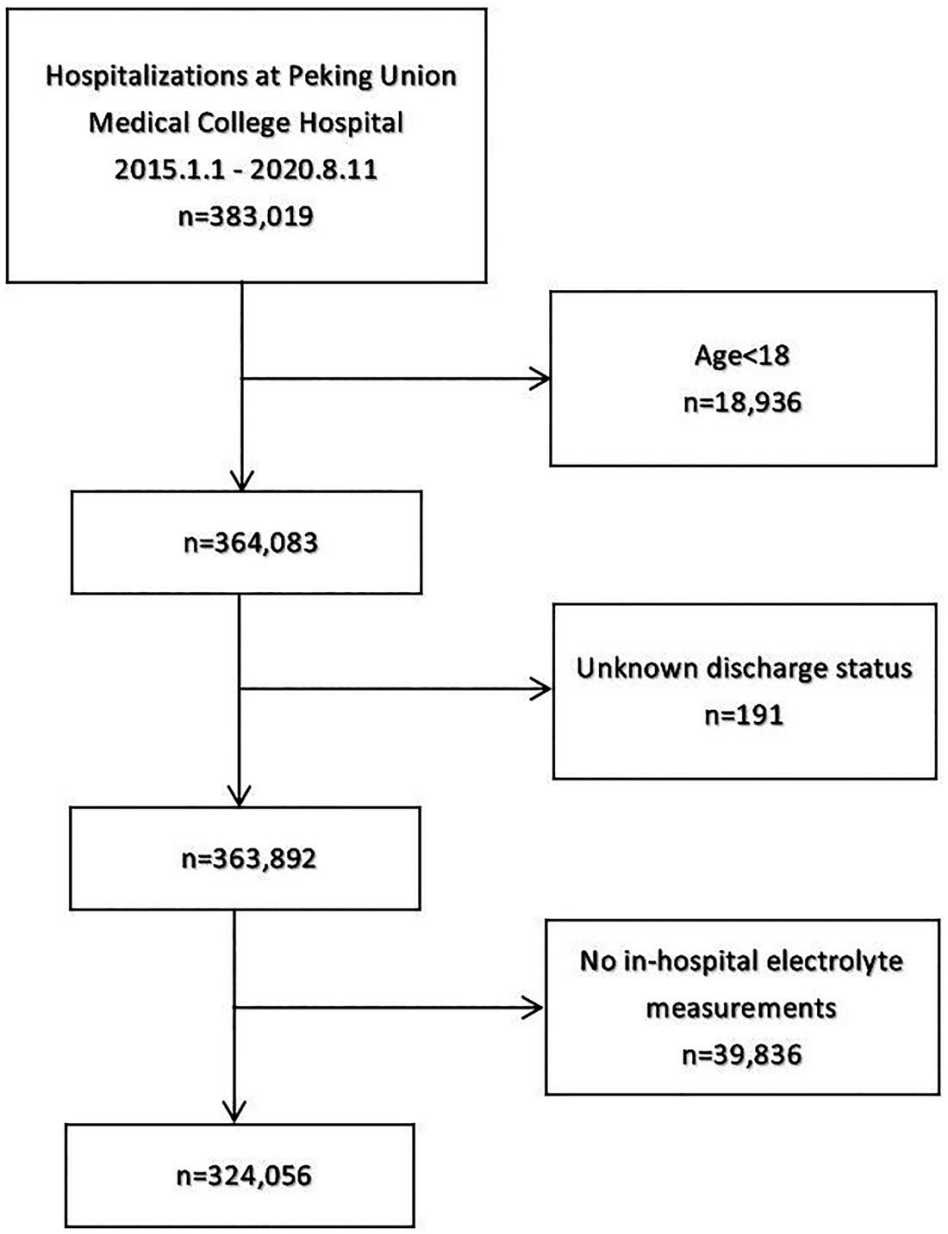
Flowchart of application of the inclusion and exclusion criteria.

This study included a total of 324,056 hospitalization records, covering 245,203 patients (Figure 1). The median age was 53 years (1st quartile, 39 years; 3rd quartile, 64 years), with 41.5% (134,326/324,056) being male; 62.1% (201,250/324,056) had no EIs, 19.1% (62,008/324,056) had 1 EI, and 18.8% (60,798/324,056) had ≥2 EIs. Patients with a greater number of EIs were more likely to be older and male, had longer hospital stays, more severe comorbidities, more severe EI and higher rates of adverse outcomes (trend P-value < 0.001). Of the eight EI types, hypokalemia (21.5% [69,628/324,056]) and hypocalcemia (16.0% [51,900/324,056]) had the highest prevalence, while hyperkalemia (0.8% [2,474/324,056]) and hypercalcemia (1.0% [3,241/324,056]) were the least common. The likelihood of severe EIs increased with number of EIs (Table 2).

**Table 2.** Characteristics of the 324,056 hospitalizations.

Characteristic	Total	No EIs	1 EI	≥2 EIs	P value
	(*n* = 324,056)	(*n* = 201,250) (62.1%)	(*n* = 62,008) (19.1%)	(*n* = 60,798) (18.8%)	for trend
Adverse outcomes	3,029 (0.9)	296 (0.1)	219 (0.4)	2,514 (4.1)	<0.001
Age, years	53 (39, 64)	51 (38, 62)	53 (40, 64)	57 (44, 67)	<0.001
Gender: male	134,326 (41.5)	81,244 (40.4)	24,789 (40.0)	28,293 (46.5)	<0.001
Duration of hospitalization, days	6 (3, 11)	5 (2, 7)	6 (4, 11)	13 (7, 22)	<0.001
CCI	2 (0, 4)	2 (0, 4)	2 (0, 4)	3 (1, 5)	<0.001
Diagnostic category					
Myocardial infarction	2,952 (0.9)	903 (0.4)	661 (1.1)	1,388 (2.3)	<0.001
Congestive heart failure	13,030 (4.0)	4,979 (2.5)	2,494 (4.0)	5,557 (9.1)	<0.001
Peripheral vascular disease	23,150 (7.1)	12,983 (6.5)	4,304 (6.9)	5,863 (9.6)	<0.001
Cerebrovascular disease	18,243 (5.6)	9,188 (4.6)	3,520 (5.7)	5,535 (9.1)	<0.001
Dementia	622 (0.2)	279 (0.1)	113 (0.2)	230 (0.4)	<0.001
Chronic lung disease	13,637 (4.2)	7,042 (3.5)	2,679 (4.3)	3,916 (6.4)	<0.001
Connective tissue disorder	14,039 (4.3)	5,204 (2.6)	2,848 (4.6)	5,987 (9.8)	<0.001
Peptic ulcer	4,034 (1.2)	1,898 (0.9)	683 (1.1)	1,453 (2.4)	<0.001
Mild liver disease	26,073 (8.0)	14,848 (7.4)	4,745 (7.7)	6,480 (10.7)	<0.001
Diabetes without complications	32,659 (10.1)	18,020 (9.0)	6,265 (10.1)	8,374 (13.8)	<0.001
Hemiplegia	552 (0.2)	203 (0.1)	98 (0.2)	251 (0.4)	<0.001
Moderate to severe renal disease	17,369 (5.4)	6,388 (3.2)	2,969 (4.8)	8,012 (13.2)	<0.001
Diabetes with complications	4,836 (1.5)	2,814 (1.4)	798 (1.3)	1,224 (2.0)	<0.001
Tumor without metastases	15,337 (4.7)	12,316 (6.1)	1,829 (2.9)	1,192 (2.0)	<0.001
Leukemia	1,854 (0.6)	626 (0.3)	347 (0.6)	881 (1.4)	<0.001
Lymphoma	7,781 (2.4)	3,889 (1.9)	1,583 (2.6)	2,309 (3.8)	<0.001
Moderate to severe liver disease	1,983 (0.6)	553 (0.3)	302 (0.5)	1,128 (1.9)	<0.001
Metastatic solid tumor	42,482 (13.1)	27,781 (13.8)	7,009 (11.3)	7,692 (12.7)	<0.001
Acquired immunodeficiency syndrome	218 (0.1)	106 (0.1)	40 (0.1)	72 (0.1)	<0.001
EI type					
Hyponatremia	29,302 (9.0)	0 (0.0)	6,742 (10.9)	22,560 (37.1)	<0.001
Severe hyponatremia	1,217 (0.4)	0 (0.0)	1 (0.0)	1,216 (2.0)	<0.001
Hypernatremia	19,633 (6.1)	0 (0.0)	4,063 (6.6)	15,570 (25.6)	<0.001
Severe hypernatremia	1,623 (0.5)	0 (0.0)	8 (0.0)	1,615 (2.7)	<0.001
Hypokalemia	69,628 (21.5)	0 (0.0)	28,513 (46.0)	41,115 (67.6)	<0.001
Severe hypokalemia	905 (0.3)	0 (0.0)	34 (0.1)	871 (1.4)	<0.001
Hyperkalemia	2,474 (0.8)	0 (0.0)	245 (0.4)	2,229 (3.7)	<0.001
Severe hyperkalemia	269 (0.1)	0 (0.0)	7 (0.0)	262 (0.4)	<0.001
Hypocalcemia	51,900 (16.0)	0 (0.0)	19,902 (32.1)	31,998 (52.6)	<0.001
Severe hypocalcemia	1,374 (0.4)	0 (0.0)	146 (0.2)	1,228 (2.0)	<0.001
Hypercalcemia	3,241 (1.0)	0 (0.0)	735 (1.2)	2,506 (4.1)	<0.001
Severe hypercalcemia	143 (0.0)	0 (0.0)	1 (0.0)	142 (0.2)	<0.001
Hypochloremia	11,192 (3.5)	0 (0.0)	468 (0.8)	10,724 (17.6)	<0.001
Severe hypochloremia	3,347 (1.0)	0 (0.0)	39 (0.1)	3,308 (5.4)	<0.001
Hyperchloremia	10,746 (3.3)	0 (0.0)	1,340 (2.2)	9,406 (15.5)	<0.001
Severe hyperchloremia	3,343 (1.0)	0 (0.0)	82 (0.1)	3,261 (5.4)	<0.001

Note: Data are presented as median (1st quartile, 3rd quartile) or frequency (percentage).

Abbreviations: CCI, Charlson comorbidity index; EI, electrolyte imbalance.

Subgroup analyses by sex and age revealed that the number of male hospitalizations peaked at around 60 years, while for females, the peak occurred at around 50 years (Fig. S1a, Table S2). The prevalence of MEIs steadily increased with age (Fig. S1b, Table S2). Overall, hypokalemia and hypocalcemia were the most prevalent EI types, whereas hyperkalemia and hypercalcemia were relatively uncommon. The prevalence of most EI types showed a gradual increase with age, followed by a marked rise around 90 years – a trend more pronounced in females than in males (Fig. S2, Table S3).

### Prevalence and association analysis of EI combinations

**Figure 2.**
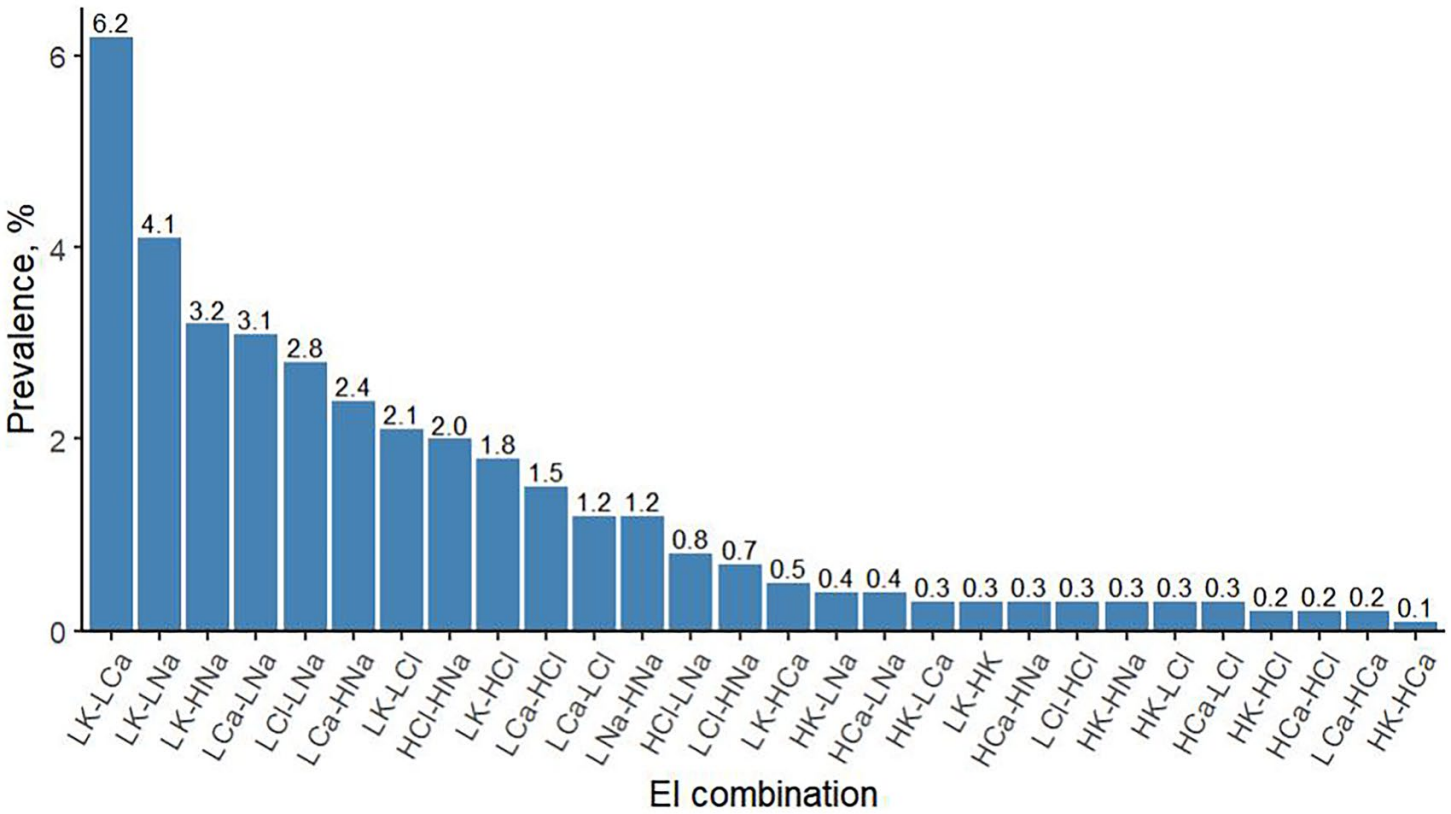
Prevalence of the 28 EI combinations. EI, electrolyte imbalance; HCa, hypercalcemia; HCl, hyperchloremia; HK, hyperkalemia; HNa, hypernatremia; LCa, hypocalcemia; LCl, hypochloremia; LK, hypokalemia; LNa, hyponatremia.

Of the 28 EI combinations, hypokalemia-hypocalcemia was the most common (6.2% [20,241/324,056]), followed by hypokalemia–hyponatremia (4.1% [13,256/324,056]), while hyperkalemia–hypercalcemia was the rarest (0.1% [375/324,056]) (Figure 2, Table 4). The strongest associations were observed for hypochloremia–hyponatremia (OR = 67.56 [95% CI: 62.37–73.28]), hyperchloremia–hypernatremia (OR = 32.28 [95% CI: 30.18–34.53]), and hyperkalemia–hypercalcemia (OR = 19.89 [95% CI: 16.48–23.84]) (Table 4).

**Table 3.** Risks of adverse outcomes for the eight EI types.

EI type	No. of hospitalizations (%)	No. of adverse outcomes (%)	Adjusted OR (95% CI)^a^	PAF (95% CI)*
Hyponatremia	29,302 (9.0)	1,511 (5.2)	2.56 (2.22, 2.95)	9.12 (7.92, 10.33)
Hypernatremia	19,633 (6.1)	1,628 (8.3)	5.73 (4.98, 6.60)	22.63 (22.02, 23.25)
Hypokalemia	69,628 (21.5)	1,693 (2.4)	1.41 (1.24, 1.59)	7.12 (4.36, 9.89)
Hyperkalemia	2,474 (0.8)	474 (19.2)	2.22 (1.82, 2.70)	0.82 (0.64, 0.99)
Hypocalcemia	51,900 (16.0)	1,258 (2.4)	1.22 (1.08, 1.38)	2.96 (0.75, 5.17)
Hypercalcemia	3,241 (1.0)	505 (15.6)	2.72 (2.24, 3.29)	1.93 (1.72, 2.15)
Hypochloremia	11,192 (3.5)	1,052 (9.4)	2.49 (2.13, 2.91)	4.24 (3.68, 4.81)
Hyperchloremia	10,746 (3.3)	935 (8.7)	1.79 (1.53, 2.08)	2.32 (1.76, 2.88)

Note: ^a^The reference group are hospitalizations without the specific EI type. Adjusted for age, gender, Charlson comorbidity index, myocardial infarction, congestive heart failure, peripheral vascular disease, central vascular disease, moderate to severe renal disease, diabetes mellitus, chronic lung disease, moderate to severe liver disease, and all other EI types. *Estimated in subset excluding repeated hospitalizations.

Abbreviations: CI, confidence interval; EI, electrolyte imbalance; OR, odds ratio; PAF, population-attributable fraction.

**Table 4.** Risks of adverse outcomes for the 28 EI combinations.

EI combination	No. of hospitalizations (%)	Unadjusted OR (95% CI)^a^	No. of adverse outcomes (%)	Adjusted OR (95% CI)*	PAF (95% CI)^b^
LNa-HNa	3,979 (1.2)	2.80 (2.64, 2.97)	710 (17.8)	13.00 (10.09, 16.74)	10.92 (10.62, 11.22)
LK-LNa	13,256 (4.1)	3.49 (3.36, 3.63)	971 (7.3)	3.45 (2.79, 4.25)	6.51 (5.63, 7.38)
LK-LCl	6,658 (2.1)	5.83 (5.48, 6.20)	701 (10.5)	3.23 (2.58, 4.03)	3.54 (3.04, 4.04)
LK-LCa	20,241 (6.2)	2.88 (2.79, 2.98)	844 (4.2)	1.68 (1.38, 2.03)	3.49 (2.15, 4.82)
LK-HNa	10,407 (3.2)	4.67 (4.46, 4.89)	1087 (10.4)	7.89 (6.46, 9.63)	17.89 (17.37, 18.41)
LK-HK	1,090 (0.3)	2.91 (2.56, 3.30)	270 (24.8)	2.21 (1.61, 3.01)	0.34 (0.21, 0.46)
LK-HCl	5,799 (1.8)	4.58 (4.31, 4.88)	686 (11.8)	2.43 (1.95, 3.03)	2.30 (1.85, 2.74)
LK-HCa	1,575 (0.5)	3.51 (3.14, 3.92)	328 (20.8)	3.00 (2.26, 3.97)	1.04 (0.88, 1.20)
LCl-LNa	9,207 (2.8)	67.56 (62.37, 73.28)	908 (9.9)	6.21 (5.22, 7.37)	9.38 (8.92, 9.84)
LCl-HNa	2,156 (0.7)	4.03 (3.73, 4.36)	523 (24.3)	11.06 (8.51, 14.34)	6.00 (5.81, 6.18)
LCa-LNa	10,016 (3.1)	3.14 (3.01, 3.27)	717 (7.2)	2.93 (2.37, 3.62)	4.03 (3.31, 4.74)
LCa-LCl	4,049 (1.2)	3.14 (2.95, 3.34)	503 (12.4)	2.78 (2.18, 3.53)	1.80 (1.46, 2.15)
LCa-HNa	7,795 (2.4)	3.89 (3.70, 4.08)	833 (10.7)	6.75 (5.50, 8.26)	12.40 (11.94, 12.86)
LCa-HCl	4,952 (1.5)	4.85 (4.56, 5.16)	533 (10.8)	2.10 (1.68, 2.63)	1.55 (1.16, 1.95)
HK-LNa	1,365 (0.4)	12.94 (11.39, 14.70)	306 (22.4)	4.19 (3.09, 5.64)	0.93 (0.78, 1.07)
HK-LCl	893 (0.3)	17.08 (14.92, 19.52)	248 (27.8)	3.84 (2.77, 5.29)	0.66 (0.56, 0.77)
HK-LCa	1,102 (0.3)	4.28 (3.77, 4.86)	247 (22.4)	2.34 (1.71, 3.18)	0.43 (0.31, 0.56)
HK-HNa	1,015 (0.3)	11.32 (9.94, 12.89)	334 (32.9)	10.23 (7.59, 13.73)	2.74 (2.64, 2.84)
HK-HCl	712 (0.2)	12.55 (10.87, 14.45)	190 (26.7)	2.72 (1.91, 3.85)	0.32 (0.23, 0.41)
HCl-LNa	2,488 (0.8)	3.22 (2.99, 3.47)	423 (17.0)	3.30 (2.55, 4.26)	1.28 (1.05, 1.51)
HCl-HNa	6,327 (2.0)	32.28 (30.18, 34.53)	888 (14.0)	10.40 (8.71, 12.41)	15.29 (14.99, 15.59)
HCa-LNa	1,302 (0.4)	7.02 (6.27, 7.86)	303 (23.3)	4.93 (3.65, 6.63)	1.29 (1.16, 1.43)
HCa-LCl	871 (0.3)	11.06 (9.72, 12.55)	248 (28.5)	4.44 (3.21, 6.12)	0.95 (0.85, 1.04)
HCa-HNa	1,086 (0.3)	8.21 (7.29, 9.24)	395 (36.4)	14.96 (11.34, 19.68)	4.93 (4.83, 5.03)
HCa-HCl	707 (0.2)	8.64 (7.53, 9.89)	171 (24.2)	3.28 (2.27, 4.71)	0.49 (0.40, 0.59)
LCa-HCa	589 (0.2)	1.17 (1.01, 1.34)	177 (30.1)	2.55 (1.75, 3.66)	0.34 (0.26, 0.42)
LCl-HCl	1,017 (0.3)	3.12 (2.79, 3.47)	253 (24.9)	2.62 (1.90, 3.60)	0.46 (0.34, 0.58)
HK-HCa	375 (0.1)	19.89 (16.48, 23.84)	134 (35.7)	3.34 (2.17, 5.10)	0.24 (0.19, 0.30)

Note: ^a^Tendency for co-occurrence between the two EIs. *The reference group are hospitalizations without the specific EI combination. Adjusted for age, gender, Charlson comorbidity index, myocardial infarction, congestive heart failure, peripheral vascular disease, central vascular disease, moderate to severe renal disease, diabetes mellitus, chronic lung disease, moderate to severe liver disease, and all other EI types. ^b^Estimated in subset excluding repeated hospitalizations.

Abbreviations: CI, confidence interval; EI, electrolyte imbalance; HCa, hypercalcemia; HCl, hyperchloremia; HK, hyperkalemia; HNa, hypernatremia; LCa, hypocalcemia; LCl, hypochloremia; LK, hypokalemia; LNa, hyponatremia; OR, odds ratio; PAF, population-attributable fraction.

### Association between number of EIs and adverse outcomes

The risk of adverse outcomes increased progressively with number of EIs. Each additional EI type was associated with a 124% higher risk of adverse outcomes (OR = 2.24 [95% CI: 2.19–2.29]). Compared to patients with no EIs, those with 1 EI and those with ≥2 EIs had significantly higher risks, with ORs of 2.18 (95% CI: 1.83–2.59) and 17.34 (95% CI: 15.32–19.62), respectively. These results were robust in the sensitivity analyses (Table S4).

### Risk of adverse outcomes by EI type (individual and population levels)

All EI types were significantly associated with adverse outcomes. Hypernatremia showed the strongest individual-level association (OR = 5.73 [95% CI: 4.98–6.60]), followed by hypercalcemia (OR = 2.72 [95% CI: 2.24–3.29]) and hyponatremia (OR = 2.56 [95% CI: 2.22–2.95]) (Table 3).

When considering both prevalence and individual risk, hypernatremia had the highest PAF (PAF = 22.63% [95% CI: 22.02%–23.25%]), followed by hyponatremia (9.12% [95% CI: 7.92%–10.33%]) and hypokalemia (7.12% [95% CI: 4.36%–9.89%]) (Table 3).

### Risk of adverse outcomes by EI combination (individual and population levels)

**Figure 3.**
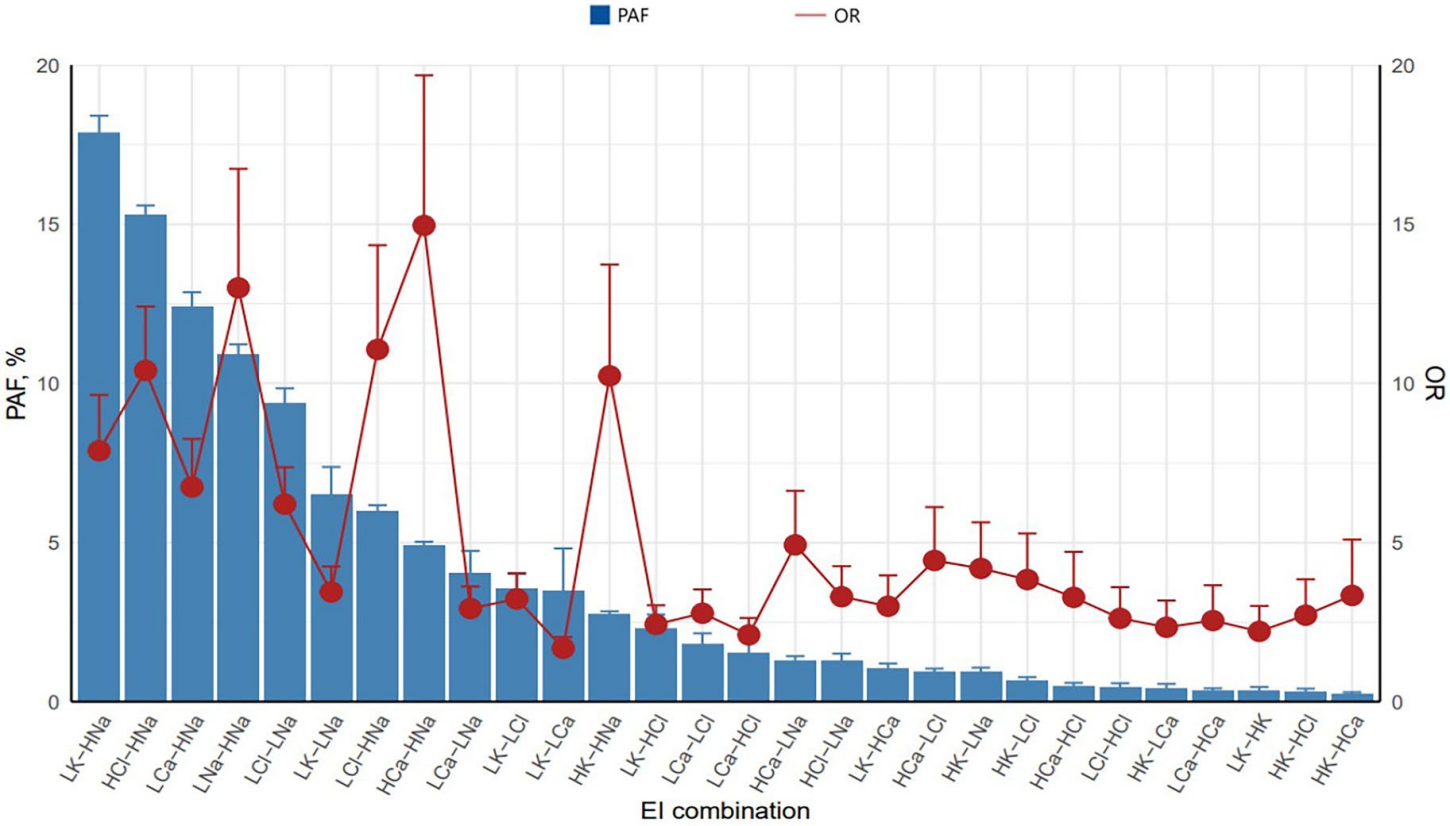
Risks of adverse outcomes for the 28 EI combinations. EI, electrolyte imbalance; HCa, hypercalcemia; HCl, hyperchloremia; HK, hyperkalemia; HNa, hypernatremia; LCa, hypocalcemia; LCl, hypochloremia; LK, hypokalemia; LNa, hyponatremia; OR, odds ratio; PAF, population-attributable fraction.

All EI combinations were significantly associated with adverse outcomes. Hypercalcemia-hypernatremia showed the strongest association (OR = 14.96 [95% CI: 11.34–19.68]), followed by hyponatremia–hypernatremia (OR = 13.00 [95% CI: 10.09–16.74]) and hypochloremia–hypernatremia (OR = 11.06 [95% CI: 8.51–14.34]) (Figure 3, Table 4).

When considering both prevalence and individual risk, hypokalemia–hypernatremia had the highest PAF (17.89% [95% CI: 17.37%–18.41%]), followed by hyperchloremia–hypernatremia (15.29% [95% CI: 14.99%–15.59%]) and hypocalcemia–hypernatremia (12.40% [95% CI: 11.94%–12.86%]) (Figure 3, Table 4).

### Interactions among EI types

Two EI combinations showed significant synergistic additive interactions: patients with both hyperchloremia and hypernatremia had an excess risk of adverse outcomes of 480% (RERI = 4.80 [95% CI: 3.05–6.55]), while those with hypercalcemia and hypernatremia had an excess risk of 698% (RERI = 6.98 [95% CI: 3.11–10.86]). These additive interactions were robust in sensitivity analyses using AP and SI. No significant synergistic multiplicative interactions were observed (Table 5).

**Table 5.** Additive and multiplicative interactions for the 28 EI combinations.

EI combination	OR10^a^	OR01^a^	OR11^a^	RERI (95% CI)	AP (95% CI)	SI (95% CI)	Multiplicative interaction (95% CI)
LNa-HNa	5.48	11.55	13.00	−3.02 (−5.64, −0.40)	−0.23 (−0.47, 0.00)	0.80 (0.63, 0.97)	0.21 (0.16, 0.27)
LK-LNa	1.91	3.78	3.45	−1.24 (−2.05, −0.44)	−0.36 (−0.62, −0.10)	0.66 (0.49, 0.83)	0.48 (0.37, 0.62)
LK-LCl	1.76	4.37	3.23	−1.91 (−2.98, −0.83)	−0.59 (−0.96, −0.22)	0.54 (0.37, 0.71)	0.42 (0.31, 0.56)
LK-LCa	1.61	1.51	1.68	−0.45 (−0.86, −0.03)	−0.27 (−0.53, 0.00)	0.60 (0.34, 0.87)	0.69 (0.53, 0.90)
LK-HNa	1.89	8.16	7.89	−1.16 (−2.80, 0.49)	−0.15 (−0.37, 0.07)	0.86 (0.67, 1.04)	0.51 (0.39, 0.67)
LK-HK	1.57	4.17	2.21	−2.54 (−3.94, −1.13)	−1.15 (−1.96, −0.34)	0.32 (0.13, 0.52)	0.34 (0.22, 0.51)
LK-HCl	1.51	2.20	2.43	−0.28 (−0.94, 0.38)	−0.11 (−0.39, 0.17)	0.84 (0.50, 1.18)	0.73 (0.54, 1.00)
LK-HCa	1.57	5.38	3.00	−2.95 (−4.83, −1.06)	−0.98 (−1.73, −0.24)	0.40 (0.20, 0.61)	0.36 (0.24, 0.54)
LCl-LNa	4.05	2.87	6.21	0.29 (−1.30, 1.88)	0.05 (−0.21, 0.30)	1.06 (0.72, 1.40)	0.53 (0.36, 0.79)
LCl-HNa	4.82	8.53	11.06	−1.29 (−3.71, 1.12)	−0.12 (−0.36, 0.12)	0.89 (0.68, 1.10)	0.27 (0.20, 0.36)
LCa-LNa	1.56	3.17	2.93	−0.80 (−1.46, −0.14)	−0.27 (−0.52, −0.02)	0.71 (0.51, 0.91)	0.59 (0.45, 0.77)
LCa-LCl	1.38	3.00	2.78	−0.61 (−1.35, 0.13)	−0.22 (−0.51, 0.07)	0.75 (0.48, 1.01)	0.67 (0.50, 0.89)
LCa-HNa	1.49	6.67	6.75	−0.41 (−1.73, 0.90)	−0.06 (−0.26, 0.14)	0.93 (0.73, 1.14)	0.68 (0.52, 0.89)
LCa-HCl	1.30	1.99	2.10	−0.19 (−0.74, 0.37)	−0.09 (−0.36, 0.18)	0.85 (0.46, 1.25)	0.81 (0.60, 1.10)
HK-LNa	4.75	2.87	4.19	−2.43 (−4.33, −0.53)	−0.58 (−1.13, −0.03)	0.57 (0.32, 0.82)	0.31 (0.20, 0.47)
HK-LCl	3.59	2.88	3.84	−1.63 (−3.16, −0.11)	−0.43 (−0.92, 0.07)	0.64 (0.35, 0.92)	0.37 (0.24, 0.57)
HK-LCa	2.74	1.28	2.34	−0.67 (−1.72, 0.38)	−0.29 (−0.79, 0.21)	0.67 (0.25, 1.09)	0.67 (0.44, 1.03)
HK-HNa	3.86	6.26	10.23	1.11 (−1.82, 4.05)	0.11 (−0.15, 0.37)	1.14 (0.77, 1.51)	0.42 (0.28, 0.65)
HK-HCl	3.11	2.01	2.72	−1.40 (−2.63, −0.18)	−0.52 (−1.09, 0.06)	0.55 (0.23, 0.87)	0.43 (0.28, 0.67)
HCl-LNa	3.12	3.66	3.30	−2.48 (−3.45, −1.51)	−0.75 (−1.16, −0.35)	0.48 (0.33, 0.64)	0.29 (0.22, 0.39)
HCl-HNa	1.19	5.41	10.40	4.80 (3.05, 6.55)	0.46 (0.35, 0.58)	2.04 (1.53, 2.56)	1.61 (0.96, 2.72)
HCa-LNa	5.59	2.93	4.93	−2.59 (−4.67, −0.51)	−0.53 (−1.04, −0.01)	0.60 (0.36, 0.85)	0.30 (0.20, 0.45)
HCa-LCl	4.48	2.98	4.44	−2.02 (−3.76, −0.27)	−0.45 (−0.95, 0.04)	0.63 (0.36, 0.90)	0.33 (0.22, 0.50)
HCa-HNa	3.12	5.86	14.96	6.98 (3.11, 10.86)	0.47 (0.32, 0.62)	2.00 (1.39, 2.61)	0.82 (0.53, 1.26)
HCa-HCl	3.60	2.02	3.28	−1.33 (−2.75, 0.09)	−0.41 (−0.95, 0.14)	0.63 (0.29, 0.97)	0.45 (0.29, 0.70)
LCa-HCa	1.29	3.23	2.55	−0.97 (−2.13, 0.19)	−0.38 (−0.94, 0.17)	0.61 (0.22, 1.01)	0.61 (0.40, 0.94)
LCl-HCl	3.40	2.45	2.62	−2.23 (−3.17, −1.28)	−0.85 (−1.39, −0.31)	0.42 (0.23, 0.62)	0.32 (0.23, 0.44)
HK-HCa	2.83	3.39	3.34	−1.88 (−3.55, −0.21)	−0.56 (−1.26, 0.13)	0.55 (0.21, 0.90)	0.35 (0.21, 0.58)

Note: ^a^The reference group are hospitalizations without the specific EI combination. Adjusted for age, gender, Charlson comorbidity index, myocardial infarction, congestive heart failure, peripheral vascular disease, central vascular disease, moderate to severe renal disease, diabetes mellitus, chronic lung disease, moderate to severe liver disease, and all other EI types. OR10, having EI1 but not EI2; OR01, having EI2 but not EI1; OR11, having both EI1 and EI2.

Abbreviations: AP, attributable proportion; CI, confidence interval; EI, electrolyte imbalance; HCa, hypercalcemia; HCl, hyperchloremia; HK, hyperkalemia; HNa, hypernatremia; LCa, hypocalcemia; LCl, hypochloremia; LK, hypokalemia; LNa, hyponatremia; OR, odds ratio; RERI, relative excess risk due to interaction; SI, synergy index.

### Stratified analyses by diagnostic category

Among patients with cancer, hyponatremia-related EI combinations represented the highest risk at both the individual and population levels (Table S5). In contrast, among patients with heart disease, moderate to severe liver disease, and moderate to severe renal disease, hypernatremia-related EI combinations consistently showed the greatest risk at both the individual and population levels (Tables S6–S8). Hyperchloremia and hypernatremia exhibited a significant synergistic additive interaction across all diagnostic categories except moderate to severe liver disease (Tables S9–S12).

## Discussion

In this retrospective study of 0.3 million hospitalizations, we applied an innovative multimorbidity framework to investigate MEIs in the general inpatient population. We revealed a high and age-progressive prevalence of MEIs, with a clear dose-response relationship between number of EIs and the risk of adverse outcomes. Hypokalemia-related EI combinations were the most common, while hypernatremia-related combinations were the most hazardous. At the individual level, hypernatremia, hypercalcemia, and hyponatremia were the primary risk factors. At the population level, hypernatremia, hypokalemia, and hyperchloremia contributed most substantially to adverse outcomes. We also identified significant synergistic additive interactions between hyperchloremia and hypernatremia, as well as between hypercalcemia and hypernatremia. Collectively, these findings provide novel insights into optimizing clinical electrolyte management.

The prevalence of MEIs reported in our study differs from earlier reports [4,5], largely due to differences in study populations. While prior research often focused on selected patient groups, our study highlights the substantial and previously underappreciated burden of MEIs within the general hospitalized population, offering a broader epidemiological perspective. Subgroup analyses revealed a higher prevalence of MEIs in older adults. Consistent with previous literature [25,26], the elderly are particularly vulnerable to EIs, likely driven by age-related physiological decline – including diminished renal function, altered hormonal regulation, and impaired thirst mechanisms – compounded by the high burden of comorbidities and polypharmacy typical in this group. Given the high frequency of MEIs among inpatients, a thorough investigation into their prognostic impact is not only warranted but clinically imperative.

In line with previous studies [4,5], we demonstrated a dose–response relationship between number of EIs and the risk of adverse outcomes. The association may be explained by two, non-mutually exclusive mechanisms. First, EIs may act as markers of underlying disease severity, wherein the primary illness is the principal driver of poor outcomes [18,27,28]. Alternatively, EIs themselves may contribute to clinical deterioration by inducing or exacerbating organ dysfunction, thereby creating a direct pathophysiological pathway to adverse events [29–31]. In our analysis, the association between number of EIs and adverse outcomes remained robust after extensive adjustment for comorbidities and other confounders. While this finding is consistent with and lends support to the hypothesis that EIs may play a more direct contributing role, we acknowledge that the observational, retrospective design of our study precludes definitive causal inference. Issues of residual confounding and uncertain temporal sequence limit our ability to establish causality. Nevertheless, the high prevalence of MEIs and their strong association with adverse outcomes highlight their importance as a critical clinical marker and a potential modifiable risk factor. This underscores the necessity for vigilant electrolyte monitoring and proactive management during hospitalization – including prevention, early detection, and timely correction – as a key component of comprehensive patient care aimed at improving outcomes.

To our knowledge, this is the first study to systematically assess the risks of different EI types and their combinations at both the individual and population levels. We observed stark contrast between the prevalence of certain EI types and their individual risks. For example, although hyperkalemia and hypercalcemia were the least prevalent EI types, their associations with adverse outcomes were significantly stronger than that of hypokalemia, which was the most common. Overall, hyper-EIs were less frequent but more harmful than hypo-EIs. This suggests that hyper-EIs may induce more severe physiological disruption and may indicate concurrent declines in kidney function, which lead to dysfunctional solute and water homeostasis [32–34]. Since PAF integrates both prevalence and individual-level risk, an EI type with a mismatch between these two components is unlikely to account for the largest share of population-level risk, either single-handedly or in combination. This also implies that the risk profiles of certain EI types or EI combinations may diverge between individual and population levels. For instance, hypernatremia and hypokalemia–hypernatremia exhibited the highest PAFs, reflecting not only the high prevalence but also the strong association with adverse outcomes. Conversely, although hyperkalemia, hypercalcemia, and hypercalcemia-hypernatremia were among the most hazardous at the individual level, their low prevalence limited their overall contribution to population-level risk.

Hypercalcemia–hypernatremia, hyponatremia–hypernatremia, and hypochloremia–hypernatremia had the strongest associations with adverse outcomes, indicating that these EI types were particularly hazardous at the individual level. In contrast, hypokalemia–hypernatremia, hyperchloremia–hypernatremia, and hypocalcemia–hypernatremia had the highest PAFs, suggesting that these EI types not only carried significant individual risks but were also prevalent. Although association does not imply clinical benefit of targeted correction, these observations carry potential implications for clinical practice and public health prioritization. At the individual level, prevention and treatment efforts should prioritize hypernatremia, hypercalcemia, and hyponatremia – particularly in light of the synergistic interaction between hypernatremia and hypercalcemia, a combination that may be encountered in the setting of malignancy [35]. From a public health perspective, greater attention should be given to hypernatremia, hypokalemia, and hyperchloremia – especially considering the synergistic interaction between hypernatremia and hyperchloremia. Clinically, these EI combinations often stem from modifiable iatrogenic factors: for instance, hypokalemia-hypernatremia may result from aggressive diuretic use without adequate electrolyte monitoring and replacement [36], while hyperchloremia-hypernatremia may be exacerbated by unbalanced fluid resuscitation, such as excessive saline administration [37]. Therefore, population-level strategies to reduce the burden of adverse outcomes could prioritize diuretic stewardship (with balanced electrolyte management), judicious fluid prescribing, and protocol-driven electrolyte surveillance in high-risk clinical settings.

**Figure 4.**
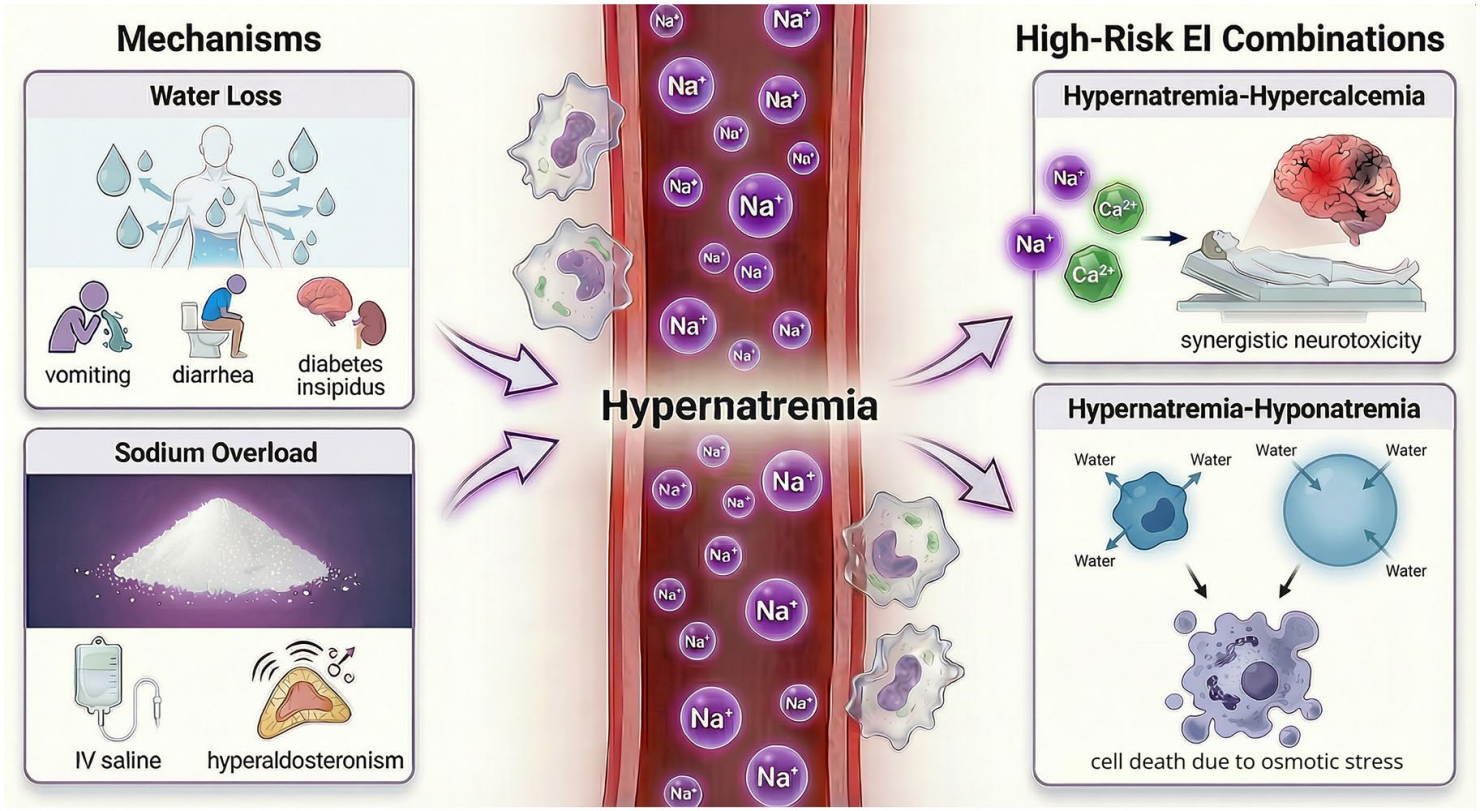
Mechanisms of hypernatremia and the related high-risk EI combinations. EI, electrolyte imbalance; IV, intravenous.

To be noteworthy, hypernatremia, due to its combination of relatively high prevalence and strong individual-level risk, was a leading contributor to adverse outcomes at both the individual and population levels. Although hypernatremia is generally less common than hyponatremia, previous studies have shown that it is more dangerous than any other EI types [18,38]. Hypernatremia and the associated hyperosmolar state can trigger a series of harmful physiological changes, including negative inotropic effects, insulin resistance, increased hepatic gluconeogenesis and impaired glucose utilization, tachypnea, cerebral cell shrinkage, and vascular rupture [39]. This EI type may also indicate dysregulation of thirst due to impaired cognition or reduced oral intake, which may further reflect more severe underlying illness and poorer overall clinical status [40]. Two hypernatremia-related combinations merit particular attention. The first combination is with hypercalcemia, which can lead to renal failure, cardiac arrhythmias (e.g. PR prolongation, QT shortening, cardiac arrest), altered mental status, and even coma [41]. Coexisting hypernatremia may exacerbate central nervous system damage, possibly explaining the high risk observed for this combination, though further investigation into the mechanism is warranted. The second combination is with hyponatremia. In line with our previous findings [42,43], wide fluctuations in serum sodium are often linked to poor prognosis. A potential mechanism is that repeated osmotic stress from sodium fluctuations disrupts cellular volume regulation, leading to DNA damage, cell cycle arrest, and apoptosis [44]. In this sense, sodium, as the major extracellular cation [45], plays a critical role in maintaining normal cellular function and viability, whereas chloride, as the main extracellular anion [46], exerts a relatively weaker effect. Prevention and correction of hypernatremia should be prioritized in clinical practice. Management strategies should address its core etiologies: water loss (e.g. vomiting, diarrhea, diabetes insipidus) and sodium overload (e.g. due to inappropriate fluid therapy, excessive sodium bicarbonate administration, hyperaldosteronism) [47](Figure 4).

A distinct pattern of electrolyte risk was observed in patients with cancer. On one hand, hyponatremia-related EI combinations were the most frequent and carried high individual and population-level risk. This suggests that hyponatremia, often caused by syndrome of inappropriate antidiuretic hormone secretion [48] or therapy-induced losses [49], may serve as an integrative marker of poor performance status and compromised nutritional state, both of which are well-established prognostic indicators in oncology [50,51]. On the other hand, hypercalcemia-hypernatremia, while less frequent, exhibited an exceptionally strong association with adverse outcomes. This combination may arise from severe hypercalcemia-induced hypotonic polyuria and dehydration, serving as a marker of advanced, refractory, and lethal malignancy [52]. Thus, both hyponatremia and hypernatremia are biologically and prognostically meaningful in cancer patients, underscoring the need for diagnosis-tailored electrolyte surveillance.

One key strength of this study is its large sample size, yet its most notable highlight is the innovative application of a multimorbidity paradigm to the study of MEIs, providing detailed and actionable guidelines for stratified electrolyte management in hospitalized patients. Nonetheless, several limitations should be acknowledged. First, due to its retrospective design, this study did not capture detailed iatrogenic factors related to EIs – such as fluid management strategies or key medication use (e.g. diuretics, chemotherapeutic agents, or immunosuppressants). The lack of these treatment data may introduce residual confounding. Future prospective studies with comprehensive therapeutic information are warranted to validate the robustness of these risk estimates. Second, our analyses were restricted to in-hospital adverse outcomes. Data on post-discharge mortality, readmission, or long-term functional status were unavailable, which precludes an assessment of the longer-term prognostic impact of MEIs. Third, as the data were obtained from a single tertiary care center, differences in disease profiles and treatment protocols across institutions may limit the generalizability of the findings. Despite this, leveraging data from a national medical center, this study has unique advantages including a long observation period, standardized laboratory testing, and consistent data quality, all of which enhance the reliability and reproducibility of the results. In the future, prognostic analyses of MEIs should be conducted across multiple centers in diverse geographic and healthcare settings to validate the external generalizability of the current findings. Incorporating long-term follow-up will also be crucial to further elucidate the sustained clinical impact of MEIs over time.

## Conclusions

This study revealed a high prevalence of MEIs among general inpatients and demonstrated a clear dose–response relationship between number of EIs and the risk of adverse outcomes. We observed that the risk profiles of different EI combinations varied between individual and population levels, with synergistic additive interactions present among certain EI types. While these findings do not prove causality, they underscore the need for strict electrolyte monitoring and management during hospitalization, and they highlight the importance of shifting from traditional single-electrolyte approaches to more integrated, multi-electrolyte strategies. At the individual level, prioritizing the management of hypernatremia, hypercalcemia, and hyponatremia may lead to the greatest improvements in patient prognosis. At the population level, hypernatremia, hypokalemia, and hyperchloremia are key intervention targets that may yield broader public health benefits. Future research can extend the methodology of this study to specific disease populations to explore the heterogeneity of MEIs epidemiology and prognosis. More importantly, prospective studies are needed to assess the effectiveness of stratified electrolyte management strategies in improving clinical outcomes.

## Supplementary Material

Supplementary Material.docx

## Data Availability

The datasets used and/or analysed during the current study are available from the corresponding author on reasonable request.
