# Supplementary material for: Multiple electrolyte imbalances in hospitalized patients: a multimorbidity perspective from a large, retrospective cohort study
Source: Ann Med. 2026 Feb 6;58(1):2618318. doi: 10.1080/07853890.2026.2618318 (PMC12884999; doi:10.1080/07853890.2026.2618318)
Supplement: Supplementary Material.docx [file IANN_A_2618318_SM6795.docx]

**Supplementary Material**

**
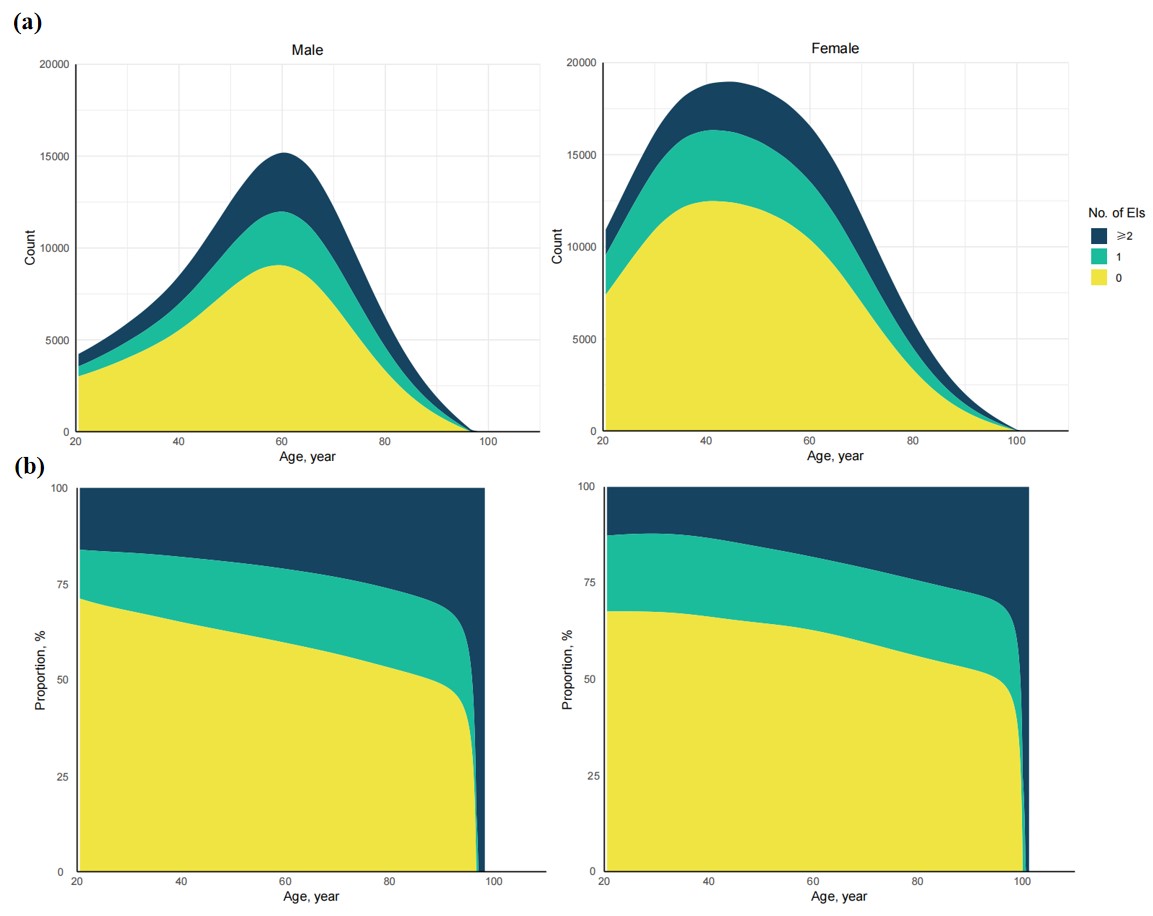
Fig. S1**

Prevalence of different numbers of EIs by sex and age subgroups: (a) Count; (b) Proportion. EIs, electrolyte imbalances.

**
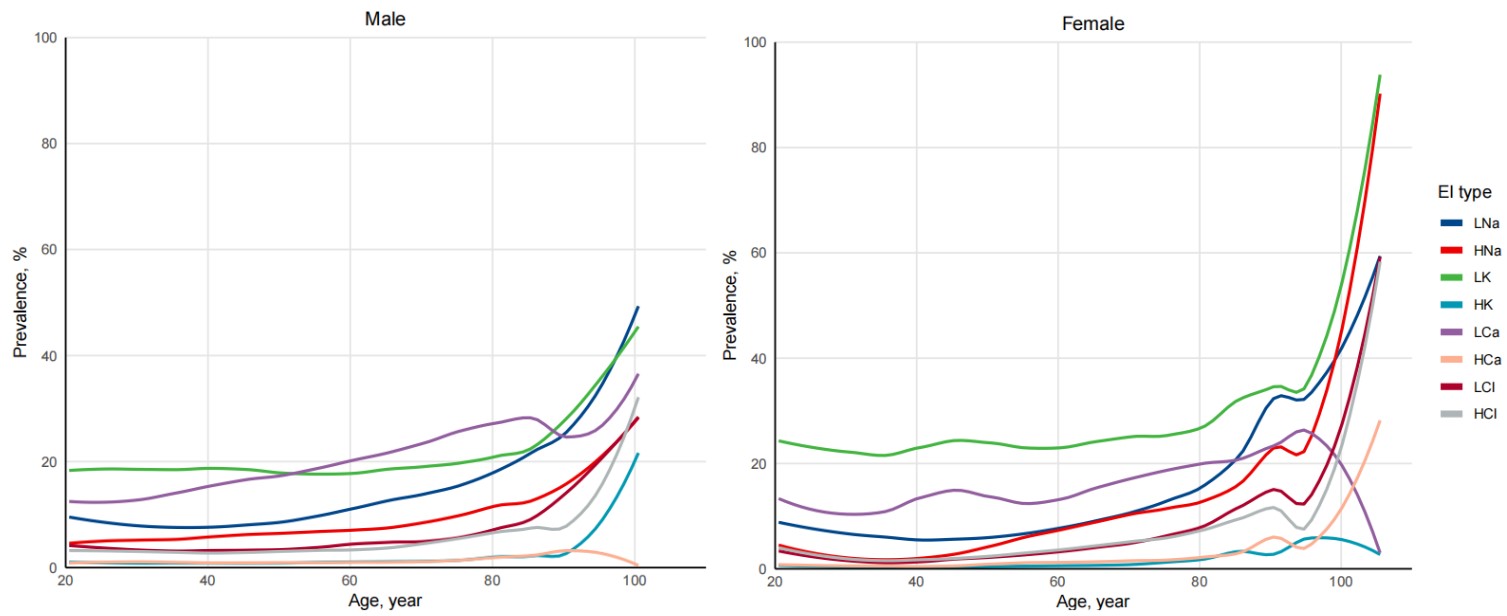
Fig. S2**

Prevalence of different EI types by sex and age subgroups. EI, electrolyte imbalance; HCa, hypercalcemia; HCl, hyperchloremia; HK, hyperkalemia; HNa, hypernatremia; LCa, hypocalcemia; LCl, hypochloremia; LK, hypokalemia; LNa, hyponatremia.

**Table S1.** Diagnostic codes in assessing Charlson comorbidity index.

| **Comorbidity** | **Points awarded** | **ICD-10 diagnostic codes** |
| --- | --- | --- |
| Myocardial infarction | 1 | I21, I22, I23 |
| Congestive heart failure | 1 | I50, I11.0, I13.0, I13.2 |
| Peripheral vascular disease | 1 | I70, I71, I72, I73, I74, I77 |
| Cerebrovascular disease | 1 | I60, I61, I62, I63, I64, I65, I66, I67, I68, I69, G45, G46 |
| Dementia | 1 | F00, F01, F02, F03, F05.1, G30 |
| Chronic pulmonary disease | 1 | J40, J41, J42, J43, J44, J45, J46, J47, J60, J61, J62, J63, J64, J65, J66, J67, J68.4, J70.1, J70.3, J84.1, J92.0, J96.1, J98.2, J98.3 |
| Connective tissue disease | 1 | M05, M06, M08, M09, M30, M31, M32, M33, M34, M35, M36, D86 |
| Ulcer disease | 1 | K22,1, K25, K26, K27, K28 |
| Mild liver disease | 1 | B18, K70.0, K70.1, K70.2, K70.3, K70.9, K71, K73, K74, K76.0 |
| Diabetes without end organ damage | 1 | E10.0, E10.1, E10.9, E11.0, E11.1, E11.9 |
| Hemiplegia | 2 | G81, G82 |
| Moderate or severe renal disease | 2 | I12, I13, N00, N01, N02, N03, N04, N05, N11, N14, N17, N18, N19, Q61 |
| Diabetes with end organ damage | 2 | E10.2, E10.3, E10.4, E10.5, E10.6, E10.7, E10.8, E11.2, E11.3, E11.4, E11.5, E11.6, E11.7, E11.8 |
| Tumor without metastasis | 2 | C0, C1, C2, C3, C4, C5, C6, C70, C71, C72, C73, C74, C75 |
| Leukemia | 2 | C91, C92, C93, C94, C95 |
| Lymphoma | 2 | C81, C82, C83, C84, C85, C88, C90, C96 |
| Moderate or severe liver disease | 3 | B15.0, B16.0, B16.2, B19.0, K70.4, K72, K76.6, I85 |
| Metastatic solid tumor | 6 | C76, C77, C78, C79, C80 |
| Acquired immune deficiency syndrome | 6 | B21, B22, B23, B24 |

Abbreviations: ICD-10, International Classification of Diseases, 10th Revision.

**Table S2.** Prevalence of different numbers of EIs by sex and age subgroups.

| **Subgroup** | **No. of hospitalizations (%)** | **No. of no EIs (%)** | **No. of 1 EI (%)** | **No. of ≥2 EIs (%)** |
| --- | --- | --- | --- | --- |
| Male (n=134,326)  [18,23)  [23,28)  [28,33)  [33,38)  [38,43)  [43,48)  [48,53)  [53,58)  [58,63)  [63,68)  [68,73)  [73,78)  [78,83)  [83,88)  [88,93)  [93,98)  [98,103)  Female (n=189,730)  [18,23)  [23,28)  [28,33)  [33,38)  [38,43)  [43,48)  [48,53)  [53,58)  [58,63)  [63,68)  [68,73)  [73,78)  [78,83)  [83,88)  [88,93)  [93,98)  [98,103)  [103,108) | 2,983 (2.2)  4,307 (3.2)  6,160 (4.6)  6,642 (4.9)  7,127 (5.3)  10,138 (7.5)  13,024 (9.7)  17,134 (12.8)  19263 (14.3)  19,804 (14.7)  12,277 (9.1)  8,232 (6.1)  4,709 (3.5)  1,931 (1.4)  498 (0.4)  79 (0.1)  18 (0.0)  3,979 (2.1)  9,288 (4.9)  18,566 (9.8)  20,725 (10.9)  17,835 (9.4)  19,762 (10.4)  19,596 (10.3)  18,932 (10.0)  18,887 (10.0)  17,632 (9.3)  10,949 (5.8)  7,293 (3.8)  4,304 (2.3)  1,508 (0.8)  389 (0.2)  66 (0.0)  16 (0.0)  3 (0.0) | 2,058 (69.0)  2,919 (67.8)  4,243 (68.9)  4,529 (68.2)  4,631 (65.0)  6,623 (65.3)  8,230 (63.2)  10,712 (62.5)  11,485 (59.6)  11,432 (57.7)  6,785 (55.3)  4,211 (51.2)  2,291 (48.7)  852 (44.1)  217 (43.6)  23 (29.1)  3 (16.7)  2,534 (63.7)  6,058 (65.2)  12,715 (68.5)  14,110 (68.1)  11,737 (65.8)  12,577 (63.6)  12,524 (63.9)  12,405 (65.5)  11,862 (62.8)  10,544 (59.8)  6,111 (55.8)  3,869 (53.1)  2,167 (50.3)  648 (43.0)  125 (32.1)  15 (22.7)  5 (31.2)  0 (0.0) | 408 (13.7)  617 (14.3)  905 (14.7)  1,021 (15.4)  1,240 (17.4)  1,688 (16.7)  2,362 (18.1)  3,175 (18.5)  3,676 (19.1)  3,975 (20.1)  2,456 (20.0)  1,804 (21.9)  972 (20.6)  387 (20.0)  86 (17.3)  14 (17.7)  3 (16.7)  716 (18.0)  1,830 (19.7)  3,736 (20.1)  4,253 (20.5)  3,730 (20.9)  4,149 (21.0)  3,793 (19.4)  3,409 (18.0)  3,399 (18.0)  3,321 (18.8)  2,217 (20.2)  1,473 (20.2)  825 (19.2)  279 (18.5)  74 (19.0)  13 (19.7)  2 (12.5)  0 (0.0) | 517 (17.3)  771 (17.9)  1,012 (16.4)  1,092 (16.4)  1,256 (17.6)  1,827 (18.0)  2,432 (18.7)  3,247 (19.0)  4,102 (21.3)  4,397 (22.2)  3,036 (24.7)  2,217 (26.9)  1,446 (30.7)  692 (35.8)  195 (39.2)  42 (53.2)  12 (66.7)  729 (18.3)  1,400 (15.1)  2,115 (11.4)  2,362 (11.4)  2,368 (13.3)  3,036 (15.4)  3,279 (16.7)  3,118 (16.5)  3,626 (19.2)  3,767 (21.4)  2,621 (23.9)  1,951 (26.8)  1,312 (30.5)  581 (38.5)  190 (48.8)  38 (57.6)  9 (56.2)  3 (100.0) |

Abbreviations: EI, electrolyte imbalance.

**Table S3.** Prevalence of different EI types by sex and age subgroups.

| **Subgroup** | **No. of hospitalizations (%)** | **No. of LNa (%)** | **No. of HNa (%)** | **No. of LK (%)** | **No. of HK (%)** | **No. of LCa (%)** | **No. of HCa (%)** | **No. of LCl (%)** | **No. of HCl (%)** |
| --- | --- | --- | --- | --- | --- | --- | --- | --- | --- |
| Male (n=134,326)  [18,23)  [23,28)  [28,33)  [33,38)  [38,43)  [43,48)  [48,53)  [53,58)  [58,63)  [63,68)  [68,73)  [73,78)  [78,83)  [83,88)  [88,93)  [93,98)  [98,103)  Female (n=189,730)  [18,23)  [23,28)  [28,33)  [33,38)  [38,43)  [43,48)  [48,53)  [53,58)  [58,63)  [63,68)  [68,73)  [73,78)  [78,83)  [83,88)  [88,93)  [93,98)  [98,103)  [103,108) | 2,983 (2.2)  4,307 (3.2)  6,160 (4.6)  6,642 (4.9)  7,127 (5.3)  10,138 (7.5)  13,024 (9.7)  17,134 (12.8)  19263 (14.3)  19,804 (14.7)  12,277 (9.1)  8,232 (6.1)  4,709 (3.5)  1,931 (1.4)  498 (0.4)  79 (0.1)  18 (0.0)  3,979 (2.1)  9,288 (4.9)  18,566 (9.8)  20,725 (10.9)  17,835 (9.4)  19,762 (10.4)  19,596 (10.3)  18,932 (10.0)  18,887 (10.0)  17,632 (9.3)  10,949 (5.8)  7,293 (3.8)  4,304 (2.3)  1,508 (0.8)  389 (0.2)  66 (0.0)  16 (0.0)  3 (0.0) | 284 (9.5)  368 (8.5)  487 (7.9)  495 (7.5)  554 (7.8)  801 (7.9)  1,145 (8.8)  1,622 (9.5)  2,197 (11.4)  2,486 (12.6)  1,725 (14.1)  1,268 (15.4)  852 (18.1)  424 (22.0)  130 (26.1)  26 (32.9)  9 (50.0)  357 (9.0)  670 (7.2)  1,275 (6.9)  1,234 (6.0)  979 (5.5)  1,085 (5.5)  1,226 (6.3)  1,202 (6.3)  1,528 (8.1)  1,601 (9.1)  1,170 (10.7)  958 (13.1)  681 (15.8)  310 (20.6)  120 (30.8)  27 (40.9)  4 (25.0)  2 (66.7) | 130 (4.4)  244 (5.7)  300 (4.9)  350 (5.3)  433 (6.1)  599 (5.9)  885 (6.8)  1,126 (6.6)  1,379 (7.2)  1,508 (7.6)  1,002 (8.2)  853 (10.4)  523 (11.1)  264 (13.7)  71 (14.3)  18 (22.8)  5 (27.8)  178 (4.5)  289 (3.1)  337 (1.8)  375 (1.8)  368 (2.1)  520 (2.6)  863 (4.4)  1,146 (6.1)  1,424 (7.5)  1,536 (8.7)  1,167 (10.7)  842 (11.5)  545 (12.7)  242 (16.0)  84 (21.6)  20 (30.3)  4 (25.0)  3 (100.0) | 539 (18.1)  831 (19.3)  1,127 (18.3)  1,210 (18.2)  1,369 (19.2)  1,839 (18.1)  2,355 (18.1)  2,988 (17.4)  3,441 (17.9)  3,656 (18.5)  2,385 (19.4)  1,589 (19.3)  1,004 (21.3)  444 (23.0)  131 (26.3)  31 (39.2)  8 (44.4)  943 (23.7)  2,270 (24.4)  4,030 (21.7)  4458 (21.5)  4,083 (22.9)  4,887 (24.7)  4,709 (24.0)  4,253 (22.5)  4,413 (23.4)  4,195 (23.8)  2,800 (25.6)  1,856 (25.4)  1,127 (26.2)  481 (31.9)  144 (37.0)  22 (33.3)  7 (43.8)  3 (100.0) | 30 (1.0)  39 (0.9)  50 (0.8)  55 (0.8)  64 (0.9)  78 (0.8)  115 (0.9)  164 (1.0)  210 (1.1)  224 (1.1)  143 (1.2)  117 (1.4)  88 (1.9)  55 (2.8)  13 (2.6)  6 (7.6)  4 (22.2)  25 (0.6)  51 (0.5)  55 (0.3)  50 (0.2)  42 (0.2)  61 (0.3)  82 (0.4)  97 (0.5)  114 (0.6)  117 (0.7)  89 (0.8)  89 (1.2)  86 (2.0)  39 (2.6)  19 (4.9)  1 (1.5)  2 (12.5)  0 (0.0) | 372 (12.5)  532 (12.4)  787 (12.8)  928 (14.0)  1,116 (15.7)  1,664 (16.4)  2,301 (17.7)  3,128 (18.3)  3,996 (20.7)  4,273 (21.6)  2,849 (23.2)  2,161 (26.3)  1,272 (27.0)  537 (27.8)  138 (27.7)  16 (20.3)  7 (38.9)  518 (13.0)  1,119 (12.0)  1,776 (9.6)  2,235 (10.8)  2,427 (13.6)  2,991 (15.1)  2,747 (14.0)  2,179 (11.5)  2,584 (13.7)  2,712 (15.4)  1,874 (17.1)  1,380 (18.9)  854 (19.8)  319 (21.2)  86 (22.1)  18 (27.3)  4 (25.0)  0 (0.0) | 26 (0.9)  42 (1.0)  68 (1.1)  70 (1.1)  55 (0.8)  99 (1.0)  121 (0.9)  153 (0.9)  193 (1.0)  211 (1.1)  122 (1.0)  124 (1.5)  84 (1.8)  48 (2.5)  13 (2.6)  3 (3.8)  0 (0.0)  33 (0.8)  54 (0.6)  110 (0.6)  108 (0.5)  79 (0.4)  101 (0.5)  180 (0.9)  229 (1.2)  223 (1.2)  232 (1.3)  174 (1.6)  123 (1.7)  88 (2.0)  51 (3.4)  17 (4.4)  6 (9.1)  0 (0.0)  1 (33.3) | 122 (4.1)  168 (3.9)  195 (3.2)  210 (3.2)  232 (3.3)  334 (3.3)  443 (3.4)  642 (3.7)  862 (4.5)  978 (4.9)  586 (4.8)  471 (5.7)  348 (7.4)  183 (9.5)  65 (13.1)  18 (22.8)  5 (27.8)  132 (3.3)  225 (2.4)  264 (1.4)  236 (1.1)  244 (1.4)  343 (1.7)  483 (2.5)  436 (2.3)  676 (3.6)  697 (4.0)  539 (4.9)  463 (6.3)  353 (8.2)  162 (10.7)  63 (16.2)  10 (15.2)  2 (12.5)  2 (66.7) | 95 (3.2)  140 (3.3)  186 (3.0)  189 (2.8)  205 (2.9)  267 (2.6)  433 (3.3)  531 (3.1)  661 (3.4)  738 (3.7)  539 (4.4)  479 (5.8)  300 (6.4)  154 (8.0)  43 (8.6)  10 (12.7)  6 (33.3)  159 (4.0)  257 (2.8)  315 (1.7)  318 (1.5)  323 (1.8)  380 (1.9)  471 (2.4)  576 (3.0)  687 (3.6)  765 (4.3)  582 (5.3)  431 (5.9)  305 (7.1)  147 (9.7)  43 (11.1)  8 (12.1)  1 (6.2)  2 (66.7) |

Abbreviations: EI, electrolyte imbalance; HCa, hypercalcemia; HCl, hyperchloremia; HK, hyperkalemia; HNa, hypernatremia; LCa, hypocalcemia; LCl, hypochloremia; LK, hypokalemia; LNa, hyponatremia.

**Table S4.** Risks of adverse outcomes by number of EIs.

| **Number of EIs** | **No. of hospitalizations (%)** | **No. of adverse outcomes (%)** | **Adjusted OR (95% CI)** | | |
| --- | --- | --- | --- | --- | --- |
|  |  |  | **Main analysis^*^** | **Sensitivity analysis:**  **Adjusting for care setting^‡^** | **Sensitivity analysis:**  **Excluding discharge against medical advice^*^** |
| No EIs | 201,250 (62.1) | 296 (0.1) | Reference | Reference | Reference |
| 1 EI | 62,008 (19.1) | 219 (0.4) | 2.18 (1.83, 2.59) | 1.96 (1.64, 2.34) | 3.75 (2.70, 5.20) |
| ≥2 EIs | 60,798 (18.8) | 2,514 (4.1) | 17.34 (15.32, 19.62) | 11.45 (10.07, 13.01) | 44.63 (34.63, 57.53) |

Note: ^*^Adjusted for age, gender, Charlson comorbidity index, myocardial infarction, congestive heart failure, peripheral vascular disease, central vascular disease, moderate to severe renal disease, diabetes mellitus, chronic lung disease, and moderate to severe liver disease. ^‡^Additionally adjusted for care setting.

Abbreviations: CI, confidence interval; EI, electrolyte imbalance; OR, odds ratio.

**Table S5.** Risks of adverse outcomes for the 28 EI combinations in patients with cancer.

| **EI combination** | **No. of hospitalizations (%)** | **Unadjusted OR (95% CI)^†^** | **No. of adverse outcomes (%)** | **Adjusted OR (95% CI)^*^** | **PAF (95% CI)^‡^** |
| --- | --- | --- | --- | --- | --- |
| LNa-HNa | 576 (1.1) | 3.15 (2.68, 3.68) | 172 (29.9) | 23.20 (12.85, 41.83) | 20.03 (19.61, 20.46) |
| LK-LNa | 2,203 (4.3) | 4.73 (4.29, 5.21) | 295 (13.4) | 8.85 (5.56, 14.11) | 25.32 (24.21, 26.43) |
| LK-LCl | 1,150 (2.3) | 6.98 (6.06, 8.03) | 207 (18.0) | 5.18 (3.25, 8.28) | 8.61 (7.74, 9.49) |
| LK-LCa | 2,812 (5.5) | 4.15 (3.80, 4.53) | 180 (6.4) | 1.56 (1.00, 2.41) | 2.98 (0.71, 5.26) |
| LK-HNa | 1,202 (2.4) | 7.33 (6.38, 8.42) | 200 (16.6) | 6.96 (4.27, 11.27) | 12.32 (11.44, 13.19) |
| LK-HK | 136 (0.3) | 4.54 (3.15, 6.53) | 55 (40.4) | 4.12 (1.87, 8.89) | 0.82 (0.62, 1.03) |
| LK-HCl | 700 (1.4) | 6.73 (5.64, 8.04) | 139 (19.9) | 3.81 (2.19, 6.61) | 3.72 (3.02, 4.42) |
| LK-HCa | 222 (0.4) | 5.80 (4.29, 7.85) | 58 (26.1) | 5.33 (2.66, 10.38) | 1.85 (1.57, 2.13) |
| LCl-LNa | 1,912 (3.7) | 93.85 (76.21, 116.74) | 295 (15.4) | 16.52 (11.47, 23.86) | 36.77 (36.22, 37.31) |
| LCl-HNa | 302 (0.6) | 3.94 (3.19, 4.82) | 110 (36.4) | 10.56 (5.78, 19.17) | 5.35 (5.04, 5.67) |
| LCa-LNa | 1,734 (3.4) | 3.02 (2.73, 3.33) | 195 (11.2) | 5.16 (3.20, 8.27) | 12.37 (11.14, 13.61) |
| LCa-LCl | 723 (1.4) | 2.92 (2.52, 3.38) | 132 (18.3) | 3.04 (1.83, 5.03) | 2.81 (2.13, 3.48) |
| LCa-HNa | 1,011 (2.0) | 5.10 (4.43, 5.86) | 134 (13.3) | 3.84 (2.28, 6.37) | 5.33 (4.42, 6.23) |
| LCa-HCl | 644 (1.3) | 5.70 (4.77, 6.80) | 92 (14.3) | 2.07 (1.14, 3.70) | 1.34 (0.62, 2.05) |
| HK-LNa | 200 (0.4) | 17.13 (11.76, 25.36) | 82 (41.0) | 18.18 (9.09, 35.93) | 6.31 (6.07, 6.54) |
| HK-LCl | 141 (0.3) | 21.12 (14.60, 30.48) | 66 (46.8) | 10.76 (5.22, 22.04) | 2.63 (2.44, 2.81) |
| HK-LCa | 141 (0.3) | 4.92 (3.42, 7.06) | 51 (36.2) | 3.12 (1.43, 6.67) | 0.58 (0.37, 0.79) |
| HK-HNa | 110 (0.2) | 13.24 (9.00, 19.26) | 57 (51.8) | 13.62 (5.98, 31.25) | 2.65 (2.48, 2.82) |
| HK-HCl | 80 (0.2) | 14.61 (9.50, 21.92) | 38 (47.5) | 7.06 (2.74, 18.07) | 0.94 (0.80, 1.08) |
| HCl-LNa | 381 (0.7) | 3.62 (2.97, 4.39) | 117 (30.7) | 11.42 (6.22, 20.86) | 7.22 (6.83, 7.61) |
| HCl-HNa | 766 (1.5) | 46.37 (38.24, 56.33) | 173 (22.6) | 12.19 (7.95, 18.61) | 14.38 (13.92, 14.85) |
| HCa-LNa | 221 (0.4) | 8.97 (6.63, 12.15) | 59 (26.7) | 14.16 (7.00, 27.78) | 5.39 (5.13, 5.66) |
| HCa-LCl | 127 (0.2) | 9.81 (6.95, 13.64) | 43 (33.9) | 8.11 (3.63, 17.39) | 1.74 (1.55, 1.92) |
| HCa-HNa | 100 (0.2) | 6.82 (4.69, 9.70) | 46 (46.0) | 15.79 (6.77, 36.56) | 2.82 (2.66, 2.97) |
| HCa-HCl | 81 (0.2) | 9.22 (6.12, 13.50) | 29 (35.8) | 8.32 (3.08, 21.60) | 1.15 (1.00, 1.30) |
| LCa-HCa | 48 (0.1) | 0.69 (0.41, 1.08) | 16 (33.3) | 2.56 (0.71, 8.92) | 0.15 (0.03, 0.26) |
| LCl-HCl | 162 (0.3) | 3.35 (2.54, 4.37) | 69 (42.6) | 5.42 (2.64, 11.03) | 1.38 (1.16, 1.60) |
| HK-HCa | 23 (0.0) | 10.29 (4.83, 19.44) | 12 (52.2) | 4.99 (1.04, 25.44) | 0.18 (0.11, 0.25) |

Note: ^†^Tendency for co-occurrence between the two EIs. ^*^The reference group are hospitalizations without the specific EI combination. Adjusted for age, gender, Charlson comorbidity index, myocardial infarction, congestive heart failure, peripheral vascular disease, central vascular disease, moderate to severe renal disease, diabetes mellitus, chronic lung disease, moderate to severe liver disease, and all other EI types. ^‡^Estimated in subset excluding repeated hospitalizations.

Abbreviations: CI, confidence interval; EI, electrolyte imbalance; HCa, hypercalcemia; HCl, hyperchloremia; HK, hyperkalemia; HNa, hypernatremia; LCa, hypocalcemia; LCl, hypochloremia; LK, hypokalemia; LNa, hyponatremia; OR, odds ratio; PAF, population-attributable fraction.

**Table S6.** Risks of adverse outcomes for the 28 EI combinations in patients with heart disease.

| **EI combination** | **No. of hospitalizations (%)** | **Unadjusted OR (95% CI)^†^** | **No. of adverse outcomes (%)** | **Adjusted OR (95% CI)^*^** | **PAF (95% CI)^‡^** |
| --- | --- | --- | --- | --- | --- |
| LNa-HNa | 847 (6.0) | 1.74 (1.50, 2.01) | 166 (19.6) | 5.23 (3.13, 8.71) | 20.20 (18.26, 22.15) |
| LK-LNa | 1,548 (10.9) | 2.27 (1.99, 2.58) | 182 (11.8) | 1.21 (0.77, 1.89) | 2.27 (0.00, 6.94) |
| LK-LCl | 1,045 (7.4) | 2.89 (2.46, 3.40) | 150 (14.4) | 1.57 (1.00, 2.45) | 4.04 (0.98, 7.09) |
| LK-LCa | 1,776 (12.6) | 2.26 (2.00, 2.56) | 178 (10.0) | 1.08 (0.73, 1.60) | 1.03 (0.00, 5.86) |
| LK-HNa | 1,626 (11.5) | 2.94 (2.57, 3.36) | 262 (16.1) | 3.89 (2.61, 5.81) | 24.96 (22.38, 27.55) |
| LK-HK | 291 (2.1) | 1.83 (1.40, 2.39) | 75 (25.8) | 1.48 (0.83, 2.59) | 0.98 (0.00, 2.13) |
| LK-HCl | 796 (5.6) | 3.44 (2.84, 4.17) | 152 (19.1) | 1.43 (0.92, 2.21) | 2.35 (0.01, 4.70) |
| LK-HCa | 329 (2.3) | 3.42 (2.56, 4.62) | 93 (28.3) | 1.97 (1.16, 3.28) | 2.20 (1.05, 3.35) |
| LCl-LNa | 1,398 (9.9) | 23.34 (19.22, 28.48) | 211 (15.1) | 2.49 (1.75, 3.52) | 12.85 (10.22, 15.47) |
| LCl-HNa | 535 (3.8) | 1.81 (1.52, 2.16) | 138 (25.8) | 6.40 (3.84, 10.62) | 16.97 (15.65, 18.29) |
| LCa-LNa | 1,163 (8.2) | 2.32 (2.02, 2.66) | 151 (13.0) | 1.52 (0.98, 2.33) | 4.09 (0.83, 7.34) |
| LCa-LCl | 668 (4.7) | 1.97 (1.67, 2.33) | 113 (16.9) | 1.99 (1.22, 3.21) | 4.48 (2.41, 6.55) |
| LCa-HNa | 1,236 (8.7) | 2.94 (2.56, 3.38) | 208 (16.8) | 4.58 (3.04, 6.86) | 23.82 (21.77, 25.87) |
| LCa-HCl | 691 (4.9) | 4.29 (3.55, 5.18) | 128 (18.5) | 1.68 (1.08, 2.61) | 3.23 (1.21, 5.24) |
| HK-LNa | 337 (2.4) | 5.54 (4.24, 7.28) | 88 (26.1) | 2.30 (1.32, 3.95) | 3.01 (1.79, 4.24) |
| HK-LCl | 265 (1.9) | 6.56 (4.99, 8.61) | 79 (29.8) | 3.19 (1.80, 5.57) | 3.95 (2.98, 4.92) |
| HK-LCa | 254 (1.8) | 2.43 (1.86, 3.17) | 66 (26.0) | 2.05 (1.15, 3.57) | 1.85 (0.87, 2.83) |
| HK-HNa | 288 (2.0) | 4.12 (3.16, 5.39) | 102 (35.4) | 7.02 (4.11, 11.86) | 10.93 (10.08, 11.78) |
| HK-HCl | 164 (1.2) | 4.45 (3.26, 6.00) | 54 (32.9) | 2.21 (1.15, 4.13) | 1.38 (0.67, 2.10) |
| HCl-LNa | 443 (3.1) | 2.16 (1.77, 2.63) | 77 (17.4) | 1.26 (0.72, 2.15) | 0.80 (0.00, 2.48) |
| HCl-HNa | 1,047 (7.4) | 29.24 (22.97, 37.63) | 215 (20.5) | 6.10 (4.24, 8.76) | 27.43 (26.02, 28.83) |
| HCa-LNa | 310 (2.2) | 6.26 (4.69, 8.41) | 85 (27.4) | 2.47 (1.41, 4.25) | 3.13 (2.00, 4.26) |
| HCa-LCl | 249 (1.8) | 7.49 (5.61, 10.01) | 77 (30.9) | 3.43 (1.92, 6.05) | 4.11 (3.18, 5.03) |
| HCa-HNa | 304 (2.2) | 6.35 (4.76, 8.51) | 122 (40.1) | 9.72 (5.91, 15.89) | 15.79 (15.04, 16.54) |
| HCa-HCl | 133 (0.9) | 3.92 (2.80, 5.42) | 47 (35.3) | 2.44 (1.21, 4.81) | 1.34 (0.71, 1.97) |
| LCa-HCa | 155 (1.1) | 1.34 (0.98, 1.81) | 51 (32.9) | 2.24 (1.15, 4.25) | 1.34 (0.64, 2.03) |
| LCl-HCl | 214 (1.5) | 1.46 (1.13, 1.87) | 48 (22.4) | 1.42 (0.71, 2.76) | 0.63 (0.00, 1.64) |
| HK-HCa | 122 (0.9) | 9.02 (6.27, 12.80) | 49 (40.2) | 3.54 (1.75, 7.02) | 2.15 (1.58, 2.72) |

Note: ^†^Tendency for co-occurrence between the two EIs. ^*^The reference group are hospitalizations without the specific EI combination. Adjusted for age, gender, Charlson comorbidity index, peripheral vascular disease, central vascular disease, moderate to severe renal disease, diabetes mellitus, chronic lung disease, moderate to severe liver disease, and all other EI types.

^‡^Estimated in subset excluding repeated hospitalizations.

Abbreviations: CI, confidence interval; EI, electrolyte imbalance; HCa, hypercalcemia; HCl, hyperchloremia; HK, hyperkalemia; HNa, hypernatremia; LCa, hypocalcemia; LCl, hypochloremia; LK, hypokalemia; LNa, hyponatremia; OR, odds ratio; PAF, population-attributable fraction.

**Table S7.** Risks of adverse outcomes for the 28 EI combinations in patients with moderate to severe renal disease.

| **EI combination** | **No. of hospitalizations (%)** | **Unadjusted OR (95% CI)^†^** | **No. of adverse outcomes (%)** | **Adjusted OR (95% CI)^*^** | **PAF (95% CI)^‡^** |
| --- | --- | --- | --- | --- | --- |
| LNa-HNa | 1,167 (6.7) | 2.06 (1.80, 2.34) | 349 (29.9) | 13.20 (8.62, 20.37) | 45.04 (44.17, 45.91) |
| LK-LNa | 2,125 (12.2) | 2.91 (2.59, 3.27) | 354 (16.7) | 1.23 (0.87, 1.74) | 2.76 (0.00, 6.80) |
| LK-LCl | 1,433 (8.3) | 3.93 (3.39, 4.56) | 276 (19.3) | 1.38 (0.96, 1.98) | 3.05 (0.23, 5.86) |
| LK-LCa | 2,142 (12.3) | 2.33 (2.08, 2.61) | 352 (16.4) | 1.21 (0.88, 1.66) | 2.51 (0.00, 6.23) |
| LK-HNa | 2,132 (12.3) | 3.83 (3.39, 4.33) | 497 (23.3) | 5.85 (4.23, 8.14) | 37.34 (35.77, 38.91) |
| LK-HK | 571 (3.3) | 1.46 (1.22, 1.75) | 152 (26.6) | 1.11 (0.72, 1.69) | 0.36 (0.00, 1.75) |
| LK-HCl | 1,431 (8.2) | 2.52 (2.20, 2.88) | 292 (20.4) | 0.98 (0.70, 1.37) | -0.14 (0.00, 2.63) |
| LK-HCa | 778 (4.5) | 3.18 (2.64, 3.83) | 187 (24.0) | 1.64 (1.12, 2.39) | 2.77 (1.17, 4.38) |
| LCl-LNa | 1,788 (10.3) | 24.46 (20.49, 29.33) | 353 (19.7) | 2.86 (2.12, 3.84) | 16.06 (13.92, 18.20) |
| LCl-HNa | 798 (4.6) | 2.52 (2.16, 2.93) | 267 (33.5) | 12.44 (8.22, 18.90) | 34.45 (33.63, 35.27) |
| LCa-LNa | 1,575 (9.1) | 2.77 (2.45, 3.13) | 294 (18.7) | 2.25 (1.59, 3.17) | 10.20 (7.68, 12.71) |
| LCa-LCl | 966 (5.6) | 2.71 (2.34, 3.14) | 218 (22.6) | 2.52 (1.71, 3.70) | 7.80 (5.98, 9.62) |
| LCa-HNa | 1,565 (9.0) | 3.33 (2.94, 3.78) | 434 (27.7) | 10.71 (7.73, 14.92) | 46.67 (45.83, 47.51) |
| LCa-HCl | 1,131 (6.5) | 2.76 (2.41, 3.17) | 252 (22.3) | 1.87 (1.33, 2.63) | 5.36 (3.37, 7.36) |
| HK-LNa | 730 (4.2) | 5.16 (4.30, 6.21) | 177 (24.2) | 2.27 (1.51, 3.39) | 5.07 (3.54, 6.61) |
| HK-LCl | 518 (3.0) | 5.41 (4.45, 6.56) | 144 (27.8) | 2.50 (1.61, 3.84) | 4.28 (3.10, 5.46) |
| HK-LCa | 580 (3.3) | 2.67 (2.22, 3.20) | 145 (25.0) | 2.18 (1.44, 3.25) | 3.78 (2.52, 5.03) |
| HK-HNa | 571 (3.3) | 3.44 (2.85, 4.13) | 211 (37.0) | 10.51 (7.07, 15.58) | 23.81 (23.06, 24.56) |
| HK-HCl | 394 (2.3) | 2.63 (2.15, 3.21) | 112 (28.4) | 1.68 (1.06, 2.61) | 1.51 (0.52, 2.50) |
| HCl-LNa | 731 (4.2) | 1.41 (1.21, 1.63) | 180 (24.6) | 1.57 (1.05, 2.33) | 2.33 (0.73, 3.93) |
| HCl-HNa | 1,589 (9.1) | 9.97 (8.63, 11.54) | 408 (25.7) | 8.65 (6.46, 11.59) | 41.16 (40.24, 42.08) |
| HCa-LNa | 671 (3.9) | 4.50 (3.74, 5.43) | 187 (27.9) | 3.29 (2.19, 4.90) | 8.11 (6.80, 9.42) |
| HCa-LCl | 515 (3.0) | 5.65 (4.64, 6.86) | 162 (31.5) | 3.73 (2.45, 5.66) | 7.49 (6.43, 8.55) |
| HCa-HNa | 668 (3.8) | 5.15 (4.28, 6.21) | 260 (38.9) | 16.36 (11.37, 23.58) | 37.14 (36.59, 37.70) |
| HCa-HCl | 353 (2.0) | 2.31 (1.87, 2.84) | 93 (26.3) | 1.97 (1.21, 3.16) | 1.93 (1.00, 2.87) |
| LCa-HCa | 367 (2.1) | 1.27 (1.04, 1.55) | 127 (34.6) | 3.07 (1.95, 4.81) | 4.19 (3.32, 5.07) |
| LCl-HCl | 356 (2.0) | 1.05 (0.87, 1.28) | 106 (29.8) | 1.46 (0.88, 2.38) | 0.93 (0.00, 1.92) |
| HK-HCa | 279 (1.6) | 4.10 (3.23, 5.17) | 105 (37.6) | 3.44 (2.11, 5.56) | 3.77 (3.05, 4.49) |

Note: ^†^Tendency for co-occurrence between the two EIs. ^*^The reference group are hospitalizations without the specific EI combination. Adjusted for age, gender, Charlson comorbidity index, myocardial infarction, congestive heart failure, peripheral vascular disease, central vascular disease, moderate to severe liver disease, diabetes mellitus, chronic lung disease, and all other EI types.

^‡^Estimated in subset excluding repeated hospitalizations.

Abbreviations: CI, confidence interval; EI, electrolyte imbalance; HCa, hypercalcemia; HCl, hyperchloremia; HK, hyperkalemia; HNa, hypernatremia; LCa, hypocalcemia; LCl, hypochloremia; LK, hypokalemia; LNa, hyponatremia; OR, odds ratio; PAF, population-attributable fraction.

**Table S8.** Risks of adverse outcomes for the 28 EI combinations in patients with moderate to severe liver disease.

| **EI combination** | **No. of hospitalizations (%)** | **Unadjusted OR (95% CI)^†^** | **No. of adverse outcomes (%)** | **Adjusted OR (95% CI)^*^** | **PAF (95% CI)^‡^** |
| --- | --- | --- | --- | --- | --- |
| LNa-HNa | 170 (8.6) | 1.47 (1.03, 2.09) | 82 (48.2) | 5.09 (2.02, 13.09) | 25.95 (21.61, 30.29) |
| LK-LNa | 388 (19.6) | 3.32 (2.43, 4.56) | 131 (33.8) | 1.86 (0.94, 3.68) | 14.37 (4.65, 24.09) |
| LK-LCl | 197 (9.9) | 2.86 (1.92, 4.31) | 88 (44.7) | 3.44 (1.53, 7.81) | 19.52 (14.33, 24.71) |
| LK-LCa | 398 (20.1) | 2.22 (1.65, 2.99) | 105 (26.4) | 1.55 (0.76, 3.17) | 9.95 (0.00, 21.44) |
| LK-HNa | 290 (14.6) | 3.20 (2.27, 4.56) | 118 (40.7) | 4.47 (2.09, 9.71) | 33.66 (28.77, 38.54) |
| LK-HK | 78 (3.9) | 1.73 (1.00, 3.01) | 48 (61.5) | 2.16 (0.80, 5.90) | 4.36 (0.82, 7.91) |
| LK-HCl | 215 (10.8) | 2.96 (2.01, 4.41) | 78 (36.3) | 1.59 (0.72, 3.49) | 6.05 (0.00, 13.51) |
| LK-HCa | 69 (3.5) | 2.05 (1.13, 3.82) | 46 (66.7) | 5.31 (1.91, 15.60) | 13.05 (10.33, 15.77) |
| LCl-LNa | 261 (13.2) | 21.15 (12.68, 37.20) | 115 (44.1) | 4.39 (2.30, 8.43) | 30.84 (26.78, 34.89) |
| LCl-HNa | 105 (5.3) | 2.03 (1.32, 3.07) | 61 (58.1) | 7.23 (2.89, 18.29) | 24.80 (22.07, 27.53) |
| LCa-LNa | 285 (14.4) | 1.80 (1.32, 2.46) | 89 (31.2) | 1.78 (0.84, 3.75) | 10.09 (1.50, 18.69) |
| LCa-LCl | 144 (7.3) | 1.69 (1.14, 2.49) | 65 (45.1) | 3.28 (1.42, 7.64) | 14.23 (9.77, 18.68) |
| LCa-HNa | 242 (12.2) | 2.56 (1.82, 3.60) | 100 (41.3) | 4.46 (2.11, 9.48) | 29.68 (25.20, 34.16) |
| LCa-HCl | 183 (9.2) | 2.52 (1.73, 3.70) | 59 (32.2) | 1.61 (0.69, 3.70) | 5.36 (0.00, 12.21) |
| HK-LNa | 87 (4.4) | 3.95 (2.27, 7.03) | 51 (58.6) | 2.33 (0.88, 6.20) | 5.51 (1.73, 9.29) |
| HK-LCl | 68 (3.4) | 6.27 (3.54, 11.08) | 44 (64.7) | 5.00 (1.82, 14.32) | 12.07 (9.37, 14.76) |
| HK-LCa | 76 (3.8) | 2.16 (1.25, 3.75) | 44 (57.9) | 2.55 (0.96, 6.88) | 5.62 (2.29, 8.94) |
| HK-HNa | 83 (4.2) | 5.75 (3.29, 10.20) | 57 (68.7) | 7.62 (2.87, 21.15) | 21.71 (19.18, 24.24) |
| HK-HCl | 56 (2.8) | 3.74 (2.09, 6.61) | 33 (58.9) | 2.45 (0.82, 7.44) | 3.94 (1.11, 6.78) |
| HCl-LNa | 109 (5.5) | 1.10 (0.73, 1.63) | 46 (42.2) | 1.62 (0.62, 4.14) | 3.27 (0.00, 8.11) |
| HCl-HNa | 224 (11.3) | 13.09 (8.58, 20.25) | 95 (42.4) | 4.99 (2.52, 9.93) | 31.08 (27.44, 34.71) |
| HCa-LNa | 64 (3.2) | 2.90 (1.59, 5.35) | 40 (62.5) | 5.01 (1.71, 14.96) | 11.47 (8.77, 14.17) |
| HCa-LCl | 49 (2.5) | 4.59 (2.44, 8.50) | 33 (67.3) | 7.16 (2.24, 24.66) | 13.21 (11.01, 15.40) |
| HCa-HNa | 78 (3.9) | 8.21 (4.40, 15.98) | 61 (78.2) | 23.87 (8.76, 72.39) | 47.36 (46.23, 48.49) |
| HCa-HCl | 29 (1.5) | 1.74 (0.83, 3.38) | 20 (69.0) | 7.15 (1.59, 37.76) | 8.26 (6.37, 10.15) |
| LCa-HCa | 41 (2.1) | 0.95 (0.50, 1.76) | 29 (70.7) | 5.61 (1.60, 22.02) | 8.70 (6.50, 10.91) |
| LCl-HCl | 44 (2.2) | 0.82 (0.46, 1.38) | 29 (65.9) | 3.07 (0.88, 11.48) | 4.38 (1.83, 6.94) |
| HK-HCa | 23 (1.2) | 3.71 (1.62, 7.81) | 19 (82.6) | 6.12 (1.17, 52.64) | 5.60 (3.76, 7.44) |

Note: ^†^Tendency for co-occurrence between the two EIs. ^*^The reference group are hospitalizations without the specific EI combination. Adjusted for age, gender, Charlson comorbidity index, myocardial infarction, congestive heart failure, peripheral vascular disease, central vascular disease, diabetes mellitus, chronic lung disease, moderate to severe renal disease, and all other EI types.

^‡^Estimated in subset excluding repeated hospitalizations.

Abbreviations: CI, confidence interval; EI, electrolyte imbalance; HCa, hypercalcemia; HCl, hyperchloremia; HK, hyperkalemia; HNa, hypernatremia; LCa, hypocalcemia; LCl, hypochloremia; LK, hypokalemia; LNa, hyponatremia; OR, odds ratio; PAF, population-attributable fraction.

**Table S9.** Additive and multiplicative interactions for the 28 EI combinations in patients with cancer.

| **EI combination** | **OR10^†^** | **OR01^†^** | **OR11^†^** | **RERI (95% CI)** | **AP (95% CI)** | **SI (95% CI)** | **Multiplicative interaction (95% CI)** |
| --- | --- | --- | --- | --- | --- | --- | --- |
| LNa-HNa | 8.23 | 9.61 | 23.20 | 6.36 (-3.80, 16.52) | 0.27 (-0.04, 0.59) | 1.40 (0.76, 2.04) | 0.29 (0.16, 0.55) |
| LK-LNa | 2.17 | 6.34 | 8.85 | 1.34 (-1.77, 4.45) | 0.15 (-0.16, 0.47) | 1.21 (0.69, 1.72) | 0.64 (0.35, 1.18) |
| LK-LCl | 2.00 | 4.27 | 5.18 | -0.08 (-2.39, 2.23) | -0.02 (-0.47, 0.43) | 0.98 (0.44, 1.52) | 0.61 (0.33, 1.12) |
| LK-LCa | 1.88 | 1.28 | 1.56 | -0.60 (-1.55, 0.36) | -0.38 (-1.04, 0.27) | 0.48 (-0.05, 1.02) | 0.65 (0.35, 1.22) |
| LK-HNa | 1.99 | 7.00 | 6.96 | -1.03 (-4.98, 2.93) | -0.15 (-0.75, 0.45) | 0.85 (0.34, 1.37) | 0.50 (0.26, 0.96) |
| LK-HK | 1.87 | 9.17 | 4.12 | -5.92 (-12.93, 1.09) | -1.44 (-3.72, 0.85) | 0.34 (-0.06, 0.74) | 0.24 (0.09, 0.66) |
| LK-HCl | 1.90 | 4.41 | 3.81 | -1.49 (-4.41, 1.43) | -0.39 (-1.24, 0.46) | 0.65 (0.14, 1.17) | 0.46 (0.22, 0.96) |
| LK-HCa | 1.66 | 3.66 | 5.33 | 1.01 (-3.57, 5.59) | 0.19 (-0.59, 0.97) | 1.31 (-0.29, 2.90) | 0.88 (0.29, 2.65) |
| LCl-LNa | 4.11 | 5.37 | 16.52 | 8.04 (1.72, 14.35) | 0.49 (0.19, 0.78) | 2.07 (0.73, 3.41) | 0.75 (0.25, 2.25) |
| LCl-HNa | 4.59 | 7.07 | 10.56 | -0.11 (-5.49, 5.28) | -0.01 (-0.52, 0.50) | 0.99 (0.43, 1.55) | 0.33 (0.17, 0.62) |
| LCa-LNa | 1.05 | 5.33 | 5.16 | -0.22 (-2.33, 1.89) | -0.04 (-0.46, 0.38) | 0.95 (0.48, 1.42) | 0.92 (0.48, 1.77) |
| LCa-LCl | 1.06 | 3.38 | 3.04 | -0.40 (-2.01, 1.21) | -0.13 (-0.69, 0.43) | 0.84 (0.23, 1.44) | 0.85 (0.45, 1.58) |
| LCa-HNa | 1.31 | 6.32 | 3.84 | -2.80 (-5.67, 0.08) | -0.73 (-1.62, 0.16) | 0.50 (0.17, 0.84) | 0.46 (0.24, 0.88) |
| LCa-HCl | 1.21 | 3.90 | 2.07 | -2.03 (-4.18, 0.11) | -0.98 (-2.23, 0.26) | 0.35 (-0.03, 0.72) | 0.44 (0.21, 0.91) |
| HK-LNa | 10.71 | 5.64 | 18.18 | 2.84 (-11.90, 17.57) | 0.16 (-0.59, 0.90) | 1.20 (0.08, 2.32) | 0.30 (0.10, 0.93) |
| HK-LCl | 6.65 | 3.39 | 10.76 | 1.72 (-7.00, 10.44) | 0.16 (-0.56, 0.88) | 1.21 (0.05, 2.38) | 0.48 (0.17, 1.35) |
| HK-LCa | 6.32 | 1.07 | 3.12 | -3.26 (-8.24, 1.71) | -1.05 (-3.08, 0.99) | 0.39 (-0.13, 0.92) | 0.46 (0.16, 1.31) |
| HK-HNa | 6.10 | 4.93 | 13.62 | 3.59 (-7.56, 14.75) | 0.26 (-0.37, 0.89) | 1.40 (0.10, 2.70) | 0.45 (0.16, 1.27) |
| HK-HCl | 5.65 | 2.86 | 7.06 | -0.46 (-7.59, 6.68) | -0.06 (-1.13, 1.00) | 0.93 (-0.15, 2.01) | 0.44 (0.14, 1.33) |
| HCl-LNa | 4.94 | 6.91 | 11.42 | 0.57 (-5.16, 6.31) | 0.05 (-0.43, 0.53) | 1.06 (0.47, 1.65) | 0.33 (0.17, 0.67) |
| HCl-HNa | 1.89 | 4.18 | 12.19 | 7.12 (2.03, 12.21) | 0.58 (0.34, 0.83) | 2.75 (0.84, 4.66) | 1.54 (0.54, 4.43) |
| HCa-LNa | 7.56 | 5.66 | 14.16 | 1.94 (-8.95, 12.84) | 0.14 (-0.57, 0.84) | 1.17 (0.14, 2.21) | 0.33 (0.11, 1.00) |
| HCa-LCl | 4.73 | 3.40 | 8.11 | 0.98 (-5.77, 7.73) | 0.12 (-0.64, 0.88) | 1.16 (0.01, 2.31) | 0.50 (0.18, 1.44) |
| HCa-HNa | 3.26 | 4.53 | 15.79 | 9.00 (-3.84, 21.85) | 0.57 (0.20, 0.94) | 2.56 (0.17, 4.94) | 1.07 (0.37, 3.13) |
| HCa-HCl | 3.43 | 2.62 | 8.32 | 3.28 (-4.75, 11.30) | 0.39 (-0.22, 1.01) | 1.81 (-0.33, 3.95) | 0.93 (0.29, 2.95) |
| LCa-HCa | 1.01 | 3.56 | 2.56 | -1.01 (-4.69, 2.66) | -0.40 (-2.26, 1.46) | 0.61 (-0.69, 1.90) | 0.71 (0.18, 2.78) |
| LCl-HCl | 3.85 | 3.48 | 5.42 | -0.91 (-4.47, 2.66) | -0.17 (-0.92, 0.58) | 0.83 (0.18, 1.48) | 0.40 (0.19, 0.86) |
| HK-HCa | 4.93 | 3.79 | 4.99 | -2.73 (-11.01, 5.54) | -0.55 (-2.99, 1.90) | 0.59 (-0.57, 1.76) | 0.27 (0.05, 1.46) |

Note: ^†^The reference group are hospitalizations without the specific EI combination. Adjusted for age, gender, Charlson comorbidity index, myocardial infarction, congestive heart failure, peripheral vascular disease, central vascular disease, moderate to severe renal disease, diabetes mellitus, chronic lung disease, moderate to severe liver disease, and all other EI types. OR10, having EI1 but not EI2; OR01, having EI2 but not EI1; OR11, having both EI1 and EI2.

Abbreviations: AP, attributable proportion; CI, confidence interval; EI, electrolyte imbalance; HCa, hypercalcemia; HCl, hyperchloremia; HK, hyperkalemia; HNa, hypernatremia; LCa, hypocalcemia; LCl, hypochloremia; LK, hypokalemia; LNa, hyponatremia; OR, odds ratio; RERI, relative excess risk due to interaction; SI, synergy index.

**Table S10.** Additive and multiplicative interactions for the 28 EI combinations in patients with heart disease.

| **EI combination** | **OR10^†^** | **OR01^†^** | **OR11^†^** | **RERI (95% CI)** | **AP (95% CI)** | **SI (95% CI)** | **Multiplicative interaction (95% CI)** |
| --- | --- | --- | --- | --- | --- | --- | --- |
| LNa-HNa | 2.99 | 7.24 | 5.23 | -4.00 (-6.79, -1.20) | -0.77 (-1.45, -0.08) | 0.51 (0.27, 0.76) | 0.24 (0.14, 0.42) |
| LK-LNa | 1.40 | 2.17 | 1.21 | -1.36 (-2.35, -0.36) | -1.12 (-2.01, -0.22) | 0.14 (-0.16, 0.43) | 0.40 (0.23, 0.68) |
| LK-LCl | 1.26 | 3.05 | 1.57 | -1.74 (-3.16, -0.32) | -1.11 (-2.12, -0.10) | 0.25 (-0.02, 0.52) | 0.41 (0.23, 0.73) |
| LK-LCa | 1.12 | 1.41 | 1.08 | -0.45 (-1.15, 0.25) | -0.42 (-1.09, 0.25) | 0.15 (-0.55, 0.86) | 0.68 (0.40, 1.18) |
| LK-HNa | 1.24 | 4.95 | 3.89 | -1.30 (-3.09, 0.50) | -0.33 (-0.83, 0.16) | 0.69 (0.36, 1.02) | 0.63 (0.37, 1.08) |
| LK-HK | 1.08 | 2.91 | 1.48 | -1.50 (-3.21, 0.20) | -1.02 (-2.42, 0.39) | 0.24 (-0.18, 0.66) | 0.47 (0.22, 1.00) |
| LK-HCl | 0.99 | 1.58 | 1.43 | -0.14 (-1.04, 0.76) | -0.10 (-0.74, 0.54) | 0.75 (-0.53, 2.03) | 0.91 (0.49, 1.71) |
| LK-HCa | 1.11 | 4.48 | 1.97 | -2.62 (-5.40, 0.17) | -1.33 (-2.97, 0.31) | 0.27 (-0.04, 0.58) | 0.40 (0.19, 0.85) |
| LCl-LNa | 2.05 | 1.44 | 2.49 | 0.01 (-1.38, 1.40) | 0.00 (-0.56, 0.56) | 1.00 (0.06, 1.94) | 0.85 (0.42, 1.72) |
| LCl-HNa | 3.23 | 5.59 | 6.40 | -1.41 (-4.22, 1.39) | -0.22 (-0.73, 0.28) | 0.79 (0.41, 1.18) | 0.36 (0.20, 0.63) |
| LCa-LNa | 1.32 | 1.56 | 1.52 | -0.37 (-1.17, 0.43) | -0.24 (-0.80, 0.31) | 0.58 (-0.08, 1.25) | 0.73 (0.42, 1.27) |
| LCa-LCl | 1.25 | 2.04 | 1.99 | -0.30 (-1.35, 0.75) | -0.15 (-0.71, 0.41) | 0.77 (0.05, 1.48) | 0.78 (0.44, 1.39) |
| LCa-HNa | 1.36 | 4.42 | 4.58 | -0.21 (-1.95, 1.53) | -0.04 (-0.43, 0.34) | 0.95 (0.50, 1.39) | 0.76 (0.44, 1.33) |
| LCa-HCl | 1.20 | 1.59 | 1.68 | -0.11 (-1.04, 0.82) | -0.06 (-0.63, 0.50) | 0.86 (-0.21, 1.94) | 0.88 (0.48, 1.63) |
| HK-LNa | 2.72 | 1.49 | 2.30 | -0.91 (-2.79, 0.98) | -0.39 (-1.32, 0.53) | 0.59 (-0.05, 1.23) | 0.57 (0.26, 1.22) |
| HK-LCl | 2.30 | 1.95 | 3.19 | -0.06 (-2.06, 1.95) | -0.02 (-0.65, 0.62) | 0.97 (0.09, 1.85) | 0.71 (0.33, 1.53) |
| HK-LCa | 2.19 | 1.20 | 2.05 | -0.34 (-1.87, 1.18) | -0.17 (-0.97, 0.64) | 0.75 (-0.20, 1.71) | 0.78 (0.37, 1.66) |
| HK-HNa | 2.72 | 4.29 | 7.02 | 1.02 (-2.54, 4.57) | 0.14 (-0.31, 0.59) | 1.20 (0.46, 1.95) | 0.60 (0.28, 1.30) |
| HK-HCl | 2.41 | 1.66 | 2.21 | -0.87 (-2.61, 0.88) | -0.39 (-1.36, 0.57) | 0.58 (-0.12, 1.28) | 0.55 (0.25, 1.22) |
| HCl-LNa | 2.67 | 2.06 | 1.26 | -2.47 (-3.81, -1.14) | -1.97 (-3.50, -0.44) | 0.09 (-0.14, 0.33) | 0.23 (0.12, 0.42) |
| HCl-HNa | 0.72 | 3.82 | 6.10 | 2.55 (0.35, 4.76) | 0.42 (0.14, 0.69) | 2.00 (0.73, 3.28) | 2.20 (0.53, 9.23) |
| HCa-LNa | 5.33 | 1.67 | 2.47 | -3.53 (-6.73, -0.32) | -1.43 (-3.04, 0.19) | 0.29 (0.01, 0.58) | 0.28 (0.13, 0.59) |
| HCa-LCl | 3.95 | 2.21 | 3.43 | -1.72 (-4.34, 0.89) | -0.50 (-1.43, 0.43) | 0.59 (0.09, 1.08) | 0.39 (0.19, 0.83) |
| HCa-HNa | 2.95 | 4.14 | 9.72 | 3.63 (-0.93, 8.19) | 0.37 (0.04, 0.70) | 1.71 (0.67, 2.76) | 0.80 (0.35, 1.80) |
| HCa-HCl | 3.27 | 1.74 | 2.44 | -1.57 (-3.69, 0.55) | -0.64 (-1.82, 0.53) | 0.48 (-0.08, 1.03) | 0.43 (0.19, 0.96) |
| LCa-HCa | 1.25 | 3.03 | 2.24 | -1.04 (-2.92, 0.84) | -0.46 (-1.50, 0.57) | 0.54 (-0.11, 1.20) | 0.59 (0.27, 1.28) |
| LCl-HCl | 2.46 | 2.07 | 1.42 | -2.11 (-3.45, -0.77) | -1.49 (-3.04, 0.07) | 0.17 (-0.19, 0.52) | 0.28 (0.14, 0.56) |
| HK-HCa | 2.31 | 2.94 | 3.54 | -0.70 (-3.43, 2.02) | -0.20 (-1.08, 0.68) | 0.78 (-0.00, 1.57) | 0.52 (0.22, 1.25) |

Note: ^†^The reference group are hospitalizations without the specific EI combination. Adjusted for age, gender, Charlson comorbidity index, peripheral vascular disease, central vascular disease, moderate to severe renal disease, diabetes mellitus, chronic lung disease, moderate to severe liver disease, and all other EI types. OR10, having EI1 but not EI2; OR01, having EI2 but not EI1; OR11, having both EI1 and EI2.

Abbreviations: AP, attributable proportion; CI, confidence interval; EI, electrolyte imbalance; HCa, hypercalcemia; HCl, hyperchloremia; HK, hyperkalemia; HNa, hypernatremia; LCa, hypocalcemia; LCl, hypochloremia; LK, hypokalemia; LNa, hyponatremia; OR, odds ratio; RERI, relative excess risk due to interaction; SI, synergy index.

**Table S11.** Additive and multiplicative interactions for the 28 EI combinations in patients with moderate to severe renal disease.

| **EI combination** | **OR10^†^** | **OR01^†^** | **OR11^†^** | **RERI (95% CI)** | **AP (95% CI)** | **SI (95% CI)** | **Multiplicative interaction (95% CI)** |
| --- | --- | --- | --- | --- | --- | --- | --- |
| LNa-HNa | 4.12 | 14.48 | 13.20 | -4.41 (-8.85, 0.04) | -0.33 (-0.73, 0.06) | 0.73 (0.50, 0.97) | 0.22 (0.14, 0.35) |
| LK-LNa | 1.02 | 2.12 | 1.23 | -0.90 (-1.66, -0.15) | -0.73 (-1.38, -0.09) | 0.20 (-0.12, 0.52) | 0.57 (0.37, 0.90) |
| LK-LCl | 0.95 | 2.58 | 1.38 | -1.15 (-2.18, -0.11) | -0.83 (-1.63, -0.03) | 0.25 (-0.04, 0.54) | 0.57 (0.35, 0.92) |
| LK-LCa | 0.80 | 1.49 | 1.21 | -0.08 (-0.62, 0.46) | -0.06 (-0.51, 0.39) | 0.73 (-0.74, 2.20) | 1.02 (0.65, 1.59) |
| LK-HNa | 1.03 | 8.40 | 5.85 | -2.58 (-4.89, -0.27) | -0.44 (-0.87, -0.02) | 0.65 (0.43, 0.88) | 0.67 (0.42, 1.07) |
| LK-HK | 0.90 | 2.21 | 1.11 | -0.99 (-1.96, -0.03) | -0.90 (-1.90, 0.11) | 0.10 (-0.31, 0.51) | 0.56 (0.32, 0.98) |
| LK-HCl | 0.78 | 1.16 | 0.98 | 0.04 (-0.45, 0.54) | 0.04 (-0.46, 0.55) | 0.28 (-4.46, 5.03) | 1.09 (0.67, 1.76) |
| LK-HCa | 0.97 | 4.23 | 1.64 | -2.57 (-4.39, -0.75) | -1.57 (-2.85, -0.29) | 0.20 (0.00, 0.39) | 0.40 (0.23, 0.69) |
| LCl-LNa | 2.21 | 1.70 | 2.86 | -0.05 (-1.34, 1.24) | -0.02 (-0.47, 0.44) | 0.97 (0.31, 1.64) | 0.76 (0.42, 1.38) |
| LCl-HNa | 4.31 | 11.25 | 12.44 | -2.12 (-6.26, 2.01) | -0.17 (-0.54, 0.20) | 0.84 (0.55, 1.14) | 0.26 (0.16, 0.41) |
| LCa-LNa | 1.93 | 2.05 | 2.25 | -0.73 (-1.63, 0.18) | -0.32 (-0.76, 0.12) | 0.63 (0.28, 0.98) | 0.57 (0.37, 0.89) |
| LCa-LCl | 1.71 | 2.25 | 2.52 | -0.44 (-1.48, 0.60) | -0.18 (-0.62, 0.27) | 0.77 (0.31, 1.24) | 0.65 (0.41, 1.05) |
| LCa-HNa | 1.68 | 7.60 | 10.71 | 2.43 (-0.31, 5.18) | 0.23 (0.01, 0.44) | 1.33 (0.92, 1.75) | 0.84 (0.53, 1.33) |
| LCa-HCl | 1.41 | 1.11 | 1.87 | 0.35 (-0.31, 1.02) | 0.19 (-0.14, 0.52) | 1.69 (-0.20, 3.58) | 1.20 (0.75, 1.92) |
| HK-LNa | 2.22 | 1.75 | 2.27 | -0.70 (-1.94, 0.54) | -0.31 (-0.92, 0.30) | 0.64 (0.15, 1.14) | 0.58 (0.33, 1.04) |
| HK-LCl | 2.06 | 2.08 | 2.50 | -0.64 (-1.91, 0.64) | -0.25 (-0.83, 0.32) | 0.70 (0.19, 1.22) | 0.58 (0.33, 1.03) |
| HK-LCa | 1.95 | 1.61 | 2.18 | -0.38 (-1.49, 0.72) | -0.18 (-0.73, 0.37) | 0.75 (0.13, 1.37) | 0.69 (0.40, 1.21) |
| HK-HNa | 2.51 | 7.91 | 10.51 | 1.09 (-2.54, 4.71) | 0.10 (-0.21, 0.42) | 1.13 (0.69, 1.57) | 0.53 (0.30, 0.94) |
| HK-HCl | 1.89 | 1.32 | 1.68 | -0.53 (-1.51, 0.45) | -0.31 (-0.99, 0.36) | 0.56 (-0.09, 1.21) | 0.67 (0.38, 1.20) |
| HCl-LNa | 1.73 | 2.15 | 1.57 | -1.31 (-2.18, -0.44) | -0.83 (-1.52, -0.15) | 0.30 (0.01, 0.59) | 0.42 (0.26, 0.67) |
| HCl-HNa | 0.74 | 6.54 | 8.65 | 2.36 (0.09, 4.64) | 0.27 (0.05, 0.49) | 1.45 (0.94, 1.96) | 1.78 (0.81, 3.91) |
| HCa-LNa | 3.46 | 1.83 | 3.29 | -1.01 (-2.70, 0.68) | -0.31 (-0.89, 0.27) | 0.69 (0.27, 1.12) | 0.52 (0.30, 0.89) |
| HCa-LCl | 3.10 | 2.15 | 3.73 | -0.52 (-2.24, 1.20) | -0.14 (-0.64, 0.36) | 0.84 (0.35, 1.34) | 0.56 (0.32, 0.97) |
| HCa-HNa | 3.51 | 7.80 | 16.36 | 6.05 (0.91, 11.19) | 0.37 (0.16, 0.58) | 1.65 (1.05, 2.25) | 0.60 (0.33, 1.06) |
| HCa-HCl | 3.26 | 1.49 | 1.97 | -1.78 (-3.14, -0.42) | -0.90 (-1.86, 0.05) | 0.35 (0.02, 0.69) | 0.41 (0.23, 0.72) |
| LCa-HCa | 1.64 | 2.84 | 3.07 | -0.40 (-1.91, 1.11) | -0.13 (-0.66, 0.40) | 0.84 (0.26, 1.41) | 0.66 (0.38, 1.14) |
| LCl-HCl | 2.39 | 1.55 | 1.46 | -1.49 (-2.45, -0.52) | -1.02 (-1.96, -0.08) | 0.24 (-0.10, 0.57) | 0.39 (0.23, 0.67) |
| HK-HCa | 1.76 | 2.58 | 3.44 | 0.10 (-1.68, 1.88) | 0.03 (-0.48, 0.54) | 1.04 (0.27, 1.81) | 0.76 (0.41, 1.41) |

Note: ^†^The reference group are hospitalizations without the specific EI combination. Adjusted for age, gender, Charlson comorbidity index, myocardial infarction, congestive heart failure, peripheral vascular disease, central vascular disease, diabetes mellitus, chronic lung disease, moderate to severe liver disease, and all other EI types. OR10, having EI1 but not EI2; OR01, having EI2 but not EI1; OR11, having both EI1 and EI2.

Abbreviations: AP, attributable proportion; CI, confidence interval; EI, electrolyte imbalance; HCa, hypercalcemia; HCl, hyperchloremia; HK, hyperkalemia; HNa, hypernatremia; LCa, hypocalcemia; LCl, hypochloremia; LK, hypokalemia; LNa, hyponatremia; OR, odds ratio; RERI, relative excess risk due to interaction; SI, synergy index.

**Table S12.** Additive and multiplicative interactions for the 28 EI combinations in patients with moderate to severe liver disease.

| **EI combination** | **OR10^†^** | **OR01^†^** | **OR11^†^** | **RERI (95% CI)** | **AP (95% CI)** | **SI (95% CI)** | **Multiplicative interaction (95% CI)** |
| --- | --- | --- | --- | --- | --- | --- | --- |
| LNa-HNa | 2.79 | 6.88 | 5.09 | -3.59 (-8.70, 1.53) | -0.70 (-1.93, 0.52) | 0.53 (0.06, 1.00) | 0.26 (0.10, 0.72) |
| LK-LNa | 1.22 | 1.44 | 1.86 | 0.19 (-1.19, 1.58) | 0.10 (-0.63, 0.84) | 1.29 (-1.33, 3.91) | 1.05 (0.39, 2.87) |
| LK-LCl | 1.54 | 4.51 | 3.44 | -1.60 (-5.58, 2.37) | -0.47 (-1.75, 0.81) | 0.60 (-0.09, 1.30) | 0.50 (0.17, 1.47) |
| LK-LCa | 1.27 | 1.26 | 1.55 | 0.03 (-1.24, 1.29) | 0.02 (-0.80, 0.83) | 1.05 (-1.46, 3.56) | 0.97 (0.36, 2.61) |
| LK-HNa | 1.87 | 6.33 | 4.47 | -2.73 (-7.57, 2.11) | -0.61 (-1.80, 0.58) | 0.56 (0.05, 1.07) | 0.38 (0.14, 1.04) |
| LK-HK | 1.35 | 2.79 | 2.16 | -0.98 (-4.29, 2.33) | -0.45 (-2.21, 1.30) | 0.54 (-0.56, 1.64) | 0.57 (0.15, 2.23) |
| LK-HCl | 1.32 | 1.51 | 1.59 | -0.23 (-1.93, 1.46) | -0.15 (-1.25, 0.95) | 0.72 (-0.92, 2.36) | 0.80 (0.25, 2.53) |
| LK-HCa | 1.33 | 6.45 | 5.31 | -1.47 (-9.98, 7.05) | -0.28 (-2.03, 1.47) | 0.75 (-0.48, 1.97) | 0.62 (0.14, 2.78) |
| LCl-LNa | 3.30 | 1.52 | 4.39 | 0.57 (-3.81, 4.94) | 0.13 (-0.84, 1.09) | 1.20 (-0.59, 2.99) | 0.87 (0.22, 3.40) |
| LCl-HNa | 5.78 | 6.18 | 7.23 | -3.72 (-10.49, 3.05) | -0.51 (-1.73, 0.70) | 0.63 (0.05, 1.20) | 0.20 (0.07, 0.59) |
| LCa-LNa | 1.50 | 1.75 | 1.78 | -0.47 (-2.07, 1.13) | -0.27 (-1.22, 0.69) | 0.62 (-0.33, 1.57) | 0.68 (0.25, 1.80) |
| LCa-LCl | 1.44 | 3.73 | 3.28 | -0.89 (-4.12, 2.34) | -0.27 (-1.37, 0.83) | 0.72 (-0.14, 1.58) | 0.61 (0.21, 1.75) |
| LCa-HNa | 1.4 | 4.13 | 4.46 | -0.06 (-3.32, 3.19) | -0.01 (-0.75, 0.72) | 0.98 (0.07, 1.90) | 0.77 (0.29, 2.09) |
| LCa-HCl | 1.25 | 1.32 | 1.61 | 0.04 (-1.47, 1.56) | 0.03 (-0.90, 0.96) | 1.08 (-1.72, 3.87) | 0.98 (0.33, 2.93) |
| HK-LNa | 3.49 | 1.67 | 2.33 | -1.83 (-5.93, 2.26) | -0.79 (-2.90, 1.33) | 0.42 (-0.36, 1.20) | 0.40 (0.10, 1.57) |
| HK-LCl | 2.54 | 3.27 | 5.00 | 0.19 (-5.11, 5.50) | 0.04 (-0.99, 1.07) | 1.05 (-0.36, 2.46) | 0.60 (0.15, 2.36) |
| HK-LCa | 2.02 | 1.23 | 2.55 | 0.30 (-2.64, 3.24) | 0.12 (-0.96, 1.20) | 1.24 (-1.38, 3.86) | 1.02 (0.26, 4.02) |
| HK-HNa | 1.97 | 3.62 | 7.62 | 3.03 (-3.95, 10.02) | 0.40 (-0.20, 0.99) | 1.84 (-0.32, 4.01) | 1.07 (0.27, 4.26) |
| HK-HCl | 2.21 | 1.36 | 2.45 | -0.11 (-3.23, 3.00) | -0.05 (-1.35, 1.26) | 0.93 (-1.00, 2.86) | 0.82 (0.20, 3.35) |
| HCl-LNa | 1.63 | 1.76 | 1.62 | -0.78 (-2.63, 1.08) | -0.48 (-1.86, 0.89) | 0.44 (-0.54, 1.42) | 0.56 (0.18, 1.71) |
| HCl-HNa | 0.71 | 3.05 | 4.99 | 2.23 (-1.06, 5.52) | 0.45 (-0.04, 0.93) | 2.26 (-0.70, 5.23) | 2.30 (0.49, 10.75) |
| HCa-LNa | 8.51 | 1.69 | 5.01 | -4.18 (-14.57, 6.20) | -0.83 (-3.36, 1.69) | 0.49 (-0.31, 1.29) | 0.35 (0.08, 1.57) |
| HCa-LCl | 8.22 | 3.70 | 7.16 | -3.75 (-14.64, 7.13) | -0.52 (-2.49, 1.44) | 0.62 (-0.30, 1.54) | 0.24 (0.05, 1.07) |
| HCa-HNa | 2.64 | 3.21 | 23.87 | 19.03 (-5.07, 43.13) | 0.80 (0.56, 1.04) | 5.95 (-1.95, 13.85) | 2.82 (0.57, 14.03) |
| HCa-HCl | 4.69 | 1.28 | 7.15 | 2.19 (-8.98, 13.36) | 0.31 (-0.84, 1.45) | 1.55 (-1.47, 4.58) | 1.19 (0.21, 6.93) |
| LCa-HCa | 1.26 | 5.14 | 5.61 | 0.22 (-7.89, 8.32) | 0.04 (-1.37, 1.45) | 1.05 (-0.82, 2.92) | 0.87 (0.18, 4.18) |
| LCl-HCl | 3.20 | 1.40 | 3.07 | -0.53 (-4.42, 3.36) | -0.17 (-1.62, 1.27) | 0.80 (-0.65, 2.24) | 0.68 (0.18, 2.65) |
| HK-HCa | 2.19 | 5.33 | 6.12 | -0.40 (-11.89, 11.09) | -0.07 (-2.05, 1.92) | 0.93 (-1.14, 2.99) | 0.53 (0.07, 4.00) |

Note: ^†^The reference group are hospitalizations without the specific EI combination. Adjusted for age, gender, Charlson comorbidity index, myocardial infarction, congestive heart failure, peripheral vascular disease, central vascular disease, diabetes mellitus, chronic lung disease, moderate to severe renal disease, and all other EI types. OR10, having EI1 but not EI2; OR01, having EI2 but not EI1; OR11, having both EI1 and EI2.

Abbreviations: AP, attributable proportion; CI, confidence interval; EI, electrolyte imbalance; HCa, hypercalcemia; HCl, hyperchloremia; HK, hyperkalemia; HNa, hypernatremia; LCa, hypocalcemia; LCl, hypochloremia; LK, hypokalemia; LNa, hyponatremia; OR, odds ratio; RERI, relative excess risk due to interaction; SI, synergy index.
